# Super-resolution expansion microscopy in plant roots

**DOI:** 10.1093/plcell/koaf006

**Published:** 2025-01-10

**Authors:** Michelle Gallei, Sven Truckenbrodt, Caroline Kreuzinger, Syamala Inumella, Vitali Vistunou, Christoph Sommer, Mojtaba R Tavakoli, Nathalie Agudelo Dueñas, Jakob Vorlaufer, Wiebke Jahr, Marek Randuch, Alexander Johnson, Eva Benková, Jiří Friml, Johann G Danzl

**Affiliations:** Institute of Science and Technology Austria, Am Campus 1, Klosterneuburg 3400, Austria; Institute of Science and Technology Austria, Am Campus 1, Klosterneuburg 3400, Austria; Institute of Science and Technology Austria, Am Campus 1, Klosterneuburg 3400, Austria; Institute of Science and Technology Austria, Am Campus 1, Klosterneuburg 3400, Austria; Institute of Science and Technology Austria, Am Campus 1, Klosterneuburg 3400, Austria; Institute of Science and Technology Austria, Am Campus 1, Klosterneuburg 3400, Austria; Institute of Science and Technology Austria, Am Campus 1, Klosterneuburg 3400, Austria; Institute of Science and Technology Austria, Am Campus 1, Klosterneuburg 3400, Austria; Institute of Science and Technology Austria, Am Campus 1, Klosterneuburg 3400, Austria; Institute of Science and Technology Austria, Am Campus 1, Klosterneuburg 3400, Austria; Institute of Science and Technology Austria, Am Campus 1, Klosterneuburg 3400, Austria; Institute of Science and Technology Austria, Am Campus 1, Klosterneuburg 3400, Austria; Institute of Science and Technology Austria, Am Campus 1, Klosterneuburg 3400, Austria; Institute of Science and Technology Austria, Am Campus 1, Klosterneuburg 3400, Austria; Institute of Science and Technology Austria, Am Campus 1, Klosterneuburg 3400, Austria

## Abstract

Super-resolution methods provide far better spatial resolution than the optical diffraction limit of about half the wavelength of light (∼200–300 nm). Nevertheless, they have yet to attain widespread use in plants, largely due to plants' challenging optical properties. Expansion microscopy (ExM) improves effective resolution by isotropically increasing the physical distances between sample structures while preserving relative spatial arrangements and clearing the sample. However, its application to plants has been hindered by the rigid, mechanically cohesive structure of plant tissues. Here, we report on whole-mount ExM of thale cress (*Arabidopsis thaliana*) root tissues (PlantEx), achieving a 4-fold resolution increase over conventional microscopy. Our results highlight the microtubule cytoskeleton organization and interaction between molecularly defined cellular constituents. Combining PlantEx with stimulated emission depletion microscopy, we increase nanoscale resolution and visualize the complex organization of subcellular organelles from intact tissues by example of the densely packed COPI-coated vesicles associated with the Golgi apparatus and put these into a cellular structural context. Our results show that ExM can be applied to increase effective imaging resolution in *Arabidopsis* root specimens.

## Teaser

The development of expansion microscopy in the Arabidopsis root enabled the imaging of molecular targets in tissues at diffraction-unlimited resolution.

## Introduction

Super-resolution optical microscopy holds tremendous promise for unravelling the molecular organization of plant cells and tissues by increasing our precision in examining biological samples, similar to the transformative power it has had in other areas of biology (Sahl et al. 2017; Sigal et al. 2018). However, the widespread adoption of super-resolution technologies in plants has been hindered by the unique physiology of plants, which makes them an optically extremely challenging system. Critically, prominent cell walls and unique organelles like vacuoles produce refractive index variations and pronounced light scattering effects (Komis et al. 2015). However, the plant community has started to overcome these challenges to advance our understanding of subcellular plant biology (Schubert 2017; Ovečka et al. 2022), with application of techniques like structured illumination microscopy (SIM) (Fitzgibbon et al. 2010; Schubert et al. 2013; Dürr et al. 2014; Komis et al. 2014; Vavrdová et al. 2019, 2020; Johnson et al. 2021), stimulated emission depletion (STED) (Kleine-Vehn et al. 2011; Sims et al. 2021) and reversible saturable optical linear fluorescence transitions (RESOLFT) (Schnorrenberg et al. 2020) microscopy, as well as single-molecule based methods including photoactivated localization microscopy (Hosy et al. 2015; Schubert and Weisshart 2015; Vavrdová et al. 2020) and (direct) stochastic optical reconstruction microscopy ((d)STORM) (Liesche et al. 2013; Dong et al. 2015; Haas et al. 2020). As much as these technologies have advanced our ability to visualize plants at improved resolution, some of these provide only an extension of resolution by a factor of 2 (linear SIM) or below (Airyscan) and hence do not access the sub-100 nm resolution range. Similarly, they are often highly susceptible to aberrations, scattering, and fluorescence background, or may require time-consuming generation of new stably expressing fluorescently labeled plant material, or equipment and expertise that is not broadly available.

Expansion microscopy (ExM) (Chen et al. 2015) is an elegant strategy to achieve effective resolution better than the diffraction limit, even when imaging with conventional, diffraction-limited microscopes. In ExM, the sample's structure is imprinted onto a swellable hydrogel that is expanded to increase physical distances between structural elements while conserving their relative arrangements, thus increasing effective resolution. In a crucial step, mechanical cohesiveness of the sample is disrupted by “mechanical homogenization,” either via proteolytic digestion (Chen et al. 2015) or denaturation (Ku et al. 2016), to abolish counter-forces and enable low-distortion, isotropic, several-fold expansion in each dimension upon washout of ions with distilled water. Critically, ExM replaces the need for high-end microscopy equipment and highly trained personnel with a sample preparation procedure of modest complexity that is readily implemented in any biological laboratory. Furthermore, expanded hydrogels are optically transparent with a homogenous refractive index, which mitigates light scattering and aberrations that often hamper other super-resolution approaches in plant samples. ExM also decreases autofluorescence, a notorious adversary in high-resolution plant imaging. Together, this combination of strengths makes ExM an ideal candidate to aid the adoption and implementation of super-resolution microscopy in the plant biology community. While there have been previous reports of applying hydrogel expansion to plant samples, they were focused on *Arabidopsis thaliana* ovules/seeds to improve antibody penetration while producing nonuniform 1.2–2.0-fold expansion (likely due to omitting the mechanical homogenization step) (Kao and Nodine 2019), were performed on isolated nuclear organelles (Kubalová et al. 2020) or isolated or single cells (Hawkins et al. 2023) rather than whole tissue, or lacked validation in terms of resolution and distortion (Hawkins et al. 2023).

**Figure 1.**
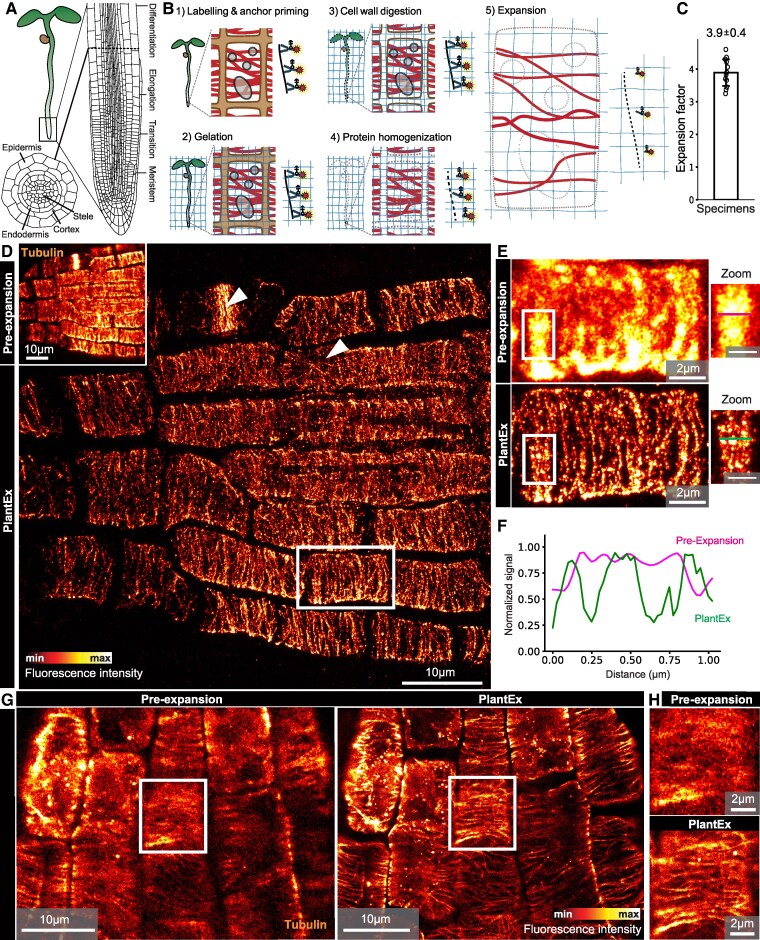
Expansion microscopy of *A. thaliana* roots. **A)**  *A. thaliana* seedling of ∼4 days age, enlarged root tip, and cross section through root, showing axial zonation and concentric layering of cells. **B)** Schematic of whole-mount expansion microscopy in plants (PlantEx) at whole specimen (left), cellular (middle), and molecular (right) levels. (1) Labeling and anchor priming. Pre-expansion immunolabeling with fluorophore (red stars) coupled with antibodies and subsequent addition of anchoring groups. Primary/secondary antibody sandwiches are shown as single antibodies for simplicity. (2) Hydrogel solution is added and anchoring groups are copolymerized into the expandable hydrogel. (3 and 4) A 2-step mechanical homogenization procedure disrupts first cell wall (Step 3) and then protein (Step 4) cohesiveness by enzymatic digestion. (5) Physical expansion increases effective resolution and renders features distinguishable that cannot be discerned without expansion. Brown shading between cells: cell walls; red filaments: labeled structures; **C)** Expansion factor for *A. thaliana* root tips, expansion factor exF = 3.9 ± 0.4 (mean ± SD, *n* = 12 independent specimens). For scaling between pre- and post-expansion data, this exF is used throughout unless otherwise noted. **D)** Confocal images of *A. thaliana* root tip immunolabeled for tubulin before expansion (inset) and with PlantEx, imaged with a high-NA objective lens in the lateral root cap. Image scaling between inset and main panel corresponds to expansion by the exF of 3.7 in this measurement. Arrowheads highlight cells of different tubulin organization. Maximum intensity projections of confocal stacks, covering approximately equal axial range in the sample, taking a 3.7-fold resolution increase in *z*-direction into account (main panel, 31 slices with 750 nm axial spacing). Scale bars: 10 µm (37 µm physical scale in the expanded sample). Scale bars refer to biological scale, i.e. original tissue size, throughout. Color bar indicates intensity lookup table with saturated pixels indicated in white. **E)** Magnified view of the region boxed in panel **D)** for pre-expansion and PlantEx images. Scale bars: 2 µm. Right: Additional zoomed views of single confocal image planes before and after expansion in regions marked on the left. Lines indicate the position of intensity profiles. Scale bars: 1 µm. **F)** Corresponding line intensity profiles in expanded and nonexpanded samples. Distance scale refers to original tissue size. **G)**, Single confocal imaging planes in the epidermal cell layer of *A. thaliana* root tip before and after expansion, using improved tubulin immunolabeling with increased labeling density. Scale bars: 10 µm (39 µm physical scale in the expanded sample, exF = 3.9). **H)**, Magnified view of the cell boxed in **G)**. Images representative of improved tubulin labeling in *n* = 4 samples.

To overcome the imaging challenges posed by plant samples and to provide an accessible super-resolution imaging methodology for plant tissues, we developed PlantEx (Fig. 1), which offers a 4-fold resolution increase in three-dimensions (3D) based on ExM, using thale cress (*A. thaliana*) root as a model system. We evaluated expansion-induced distortions and combined PlantEx with STED microscopy (Hell and Wichmann 1994; Klar et al. 2000; Gao et al. 2018; Gambarotto et al. 2019) to showcase the road toward molecular-scale optical imaging resolution in situ in plant tissues and used comprehensive structural labeling to place super-resolved molecular information into cellular context.

## Results

### Establishing ExM for super-resolution imaging of *Arabidopsis* root tissues, cells, and subcellular organelles

Given the success and wide application of ExM to a range of biological systems, we reasoned that ExM would be adoptable to plants if the specific challenges associated with plant anatomy were addressed. In particular, the mechanical cohesiveness of cell walls is expected to hinder expansion if not properly removed, as it is well established that samples with mechanically tough constituents, like, e.g. the roundworm *Caenorhabditis elegans* (Park et al. 2019; Yu et al. 2020), require specialized protocols for ExM. Even the highly complex but mechanically soft mammalian brain tissue is more challenging to expand homogeneously than monolayer cell cultures and species-dependent differences in composition of thin bacterial cell walls impact expansion (Lim et al. 2019). Similarly, tough cuticles of fruit fly (*Drosophila melanogaster)* larvae required a multi-day digestion protocol for expansion (Jiang et al. 2018). *A. thaliana* cell walls are composed of different polysaccharides, mainly cellulose, hemicellulose, and pectins (Derbyshire et al. 2007), and range in thickness from 50 nm to 1 µm, depending on tissue. We established a protocol, which overcomes the plant-specific challenges and allows *Arabidopsis* seedlings to be successfully expanded.

We focused the development of PlantEx on the *A. thaliana* root organ, as it is a major standard plant species model and the root organ constitutes a popular and fundamental model organ for plant developmental and cell biological studies (Scheres and Wolkenfelt 1998). *A. thaliana* seedlings (Fig. 1A) have a primary root featuring axial zonation (Verbelen et al. 2006) starting at the root tip with the meristematic zone of dividing cells, where also the stem cells reside, and transitioning at the shoot-to-root junction to the hypocotyl-bearing cotyledons. Similarly, root cell files are arranged in concentric layers, encompassing lateral root cap, epidermis, cortex, endodermis, and central cylinder tissue (Lee et al. 2013). Each zone has significant physiological roles in development, growth, and regeneration. Intricate spatial organization from tissue to subcellular, organelle, and macromolecular levels mirrors important roles in vital processes, such as the gravitropic response and extensive transport with nutrient uptake and synthetic activity.

We based our protocol for PlantEx (Fig. 1B) on protein-retention ExM (Chozinski et al. 2016; Tillberg et al. 2016), as here, hydrogel embedding and expansion steps are simply added after immunolabeling of specific targets. Importantly, PlantEx can be used with common reporter strategies, including the vast array of plant lines expressing fluorescent protein (FP) fusion proteins. While specialized protocols have been optimized for mild mechanical homogenization to conserve FP fluorescence (Chozinski et al. 2016; Tillberg et al. 2016), we chose to detect FP location and boost signal with fluorophore-conjugated antibodies, which avoided restrictions in designing mechanical homogenization procedures for overcoming cell wall cohesiveness.

Thus, we adopted manual and robotic whole-mount plant immunostaining protocols (Friml et al. 2003; Sauer et al. 2006; Pasternak et al. 2015; Vavrdová et al. 2019), using seedlings aged 3–4 days. These protocols differ from routine mammalian cell labeling protocols as, beyond the standard steps of chemical fixation and membrane permeabilization, plant immunolabeling requires a (mild) cell wall digestion step permitting antibody access while preserving histoarchitecture. After immunostaining, we incubated whole seedlings with Acryloyl-X solution. Acryloyl-X contains functional groups that covalently bind proteins to the hydrogel matrix, including those to be localized (labeled antibodies, for example). We subsequently cast a sodium acrylate/acrylamide/N,N′-methylenebisacrylamide-based gel designed for 4-fold expansion (Chen et al. 2015; Tillberg et al. 2016) onto the sample and allowed it to polymerize. We then applied a 2-step mechanical homogenization procedure. The 1st was plant specific and optimized for removing mechanical constraints by cell walls. We found a combination of cell-wall-component-specific enzymes — driselase, cellulase, macerozyme, pectolyase, and xylanase — effective at allowing isotropic expansion of entire root tips. The 2nd homogenization step was akin to homogenization procedures for mammalian tissues to dissolve cohesiveness from protein interactions and relied on proteinase K, a promiscuous proteolytic enzyme. Notably, the plant-specific cell-wall digestion cocktail also displayed promiscuous proteolytic activity (Supplementary Fig. S1). It thus complemented proteinase K activity for this purpose, which needs to be taken into account when balancing fluorophore retention against the degree of mechanical homogenization (Truckenbrodt et al. 2019). Immersing the sample/hydrogel hybrid in deionized water resulted in isotropic 3D expansion while conserving overall sample structure.

To demonstrate how PlantEx can aid the understanding of plant tissue architecture down to the subcellular level and to quantify its performance, we focused our super-resolution imaging on the meristematic regions of roots. We immunostained *A. thaliana* seedlings for tubulin and compared confocal pre-expansion and PlantEx images of the same region obtained with high-numerical-aperture objective lenses. Microtubules with their continuous filamentous structure and well-characterized organization were a useful first target to assess PlantEx performance in terms of impact on tissue structure and imaging outcome. Hydrogel expansion conserved cell and tissue morphologies, and, as expected from ∼4-fold expansion, a substantial resolution increase was evident with PlantEx (Fig. 1D and F, expansion factor, exF = 3.7). We determined the expansion factor as the scale factor between pre- and post-expansion datasets (Supplementary Fig. S2) and observed a mean exF = 3.9 ± 0.4 (mean ± standard deviation (SD)) when evaluating across multiple specimens (Fig. 1C, *n* = 12 independent specimens). Effective resolution increase equivalent to the exF allowed us to visualize the tubulin organizational states in different cells of the tissue at substantially increased detail over pre-expansion images (Fig. 1D and E). With PlantEx, line intensity profiles over the same structure showed separated features that coalesced in pre-expansion images (Fig. 1F), demonstrating improved resolution with PlantEx. At such high resolution, spatial sampling of the biological structures with fluorophores is a critical factor that may become limiting. In the PlantEx data in Fig. 1D and E, the discrete nature of the tubulin staining was apparent, whereas it was concealed in pre-expansion images. Effective labeling density after expansion depends on factors including the efficiency of retention of fluorophore-bearing antibody fragments in the hydrogel (Chozinski et al. 2016; Tillberg et al. 2016) and potential loss of fluorophores due to radical generation during polymerization (Truckenbrodt et al. 2019), but equally also on initial antibody penetration into the nonexpanded tissue and labeling density achieved before expansion. Therefore, we adopted an immunolabeling protocol specifically optimized for tubulin (Vavrdová et al. 2019) and observed improved fluorophore coverage of individual tubulin strands (Fig. 1G and H). This showcases that PlantEx, just as other ExM approaches, is a modular technique and improvements in one aspect often directly translate into improved overall outcome. The effect of 3D-resolution enhancement with PlantEx is particularly evident for densely packed structures. When imaging mitotic spindles (Supplementary Fig. S3), they appeared as largely homogeneous bright bars before expansion, whereas the organization of tubulin strands could be visualized with PlantEx.

### Characterization of distortions

**Figure 2.**
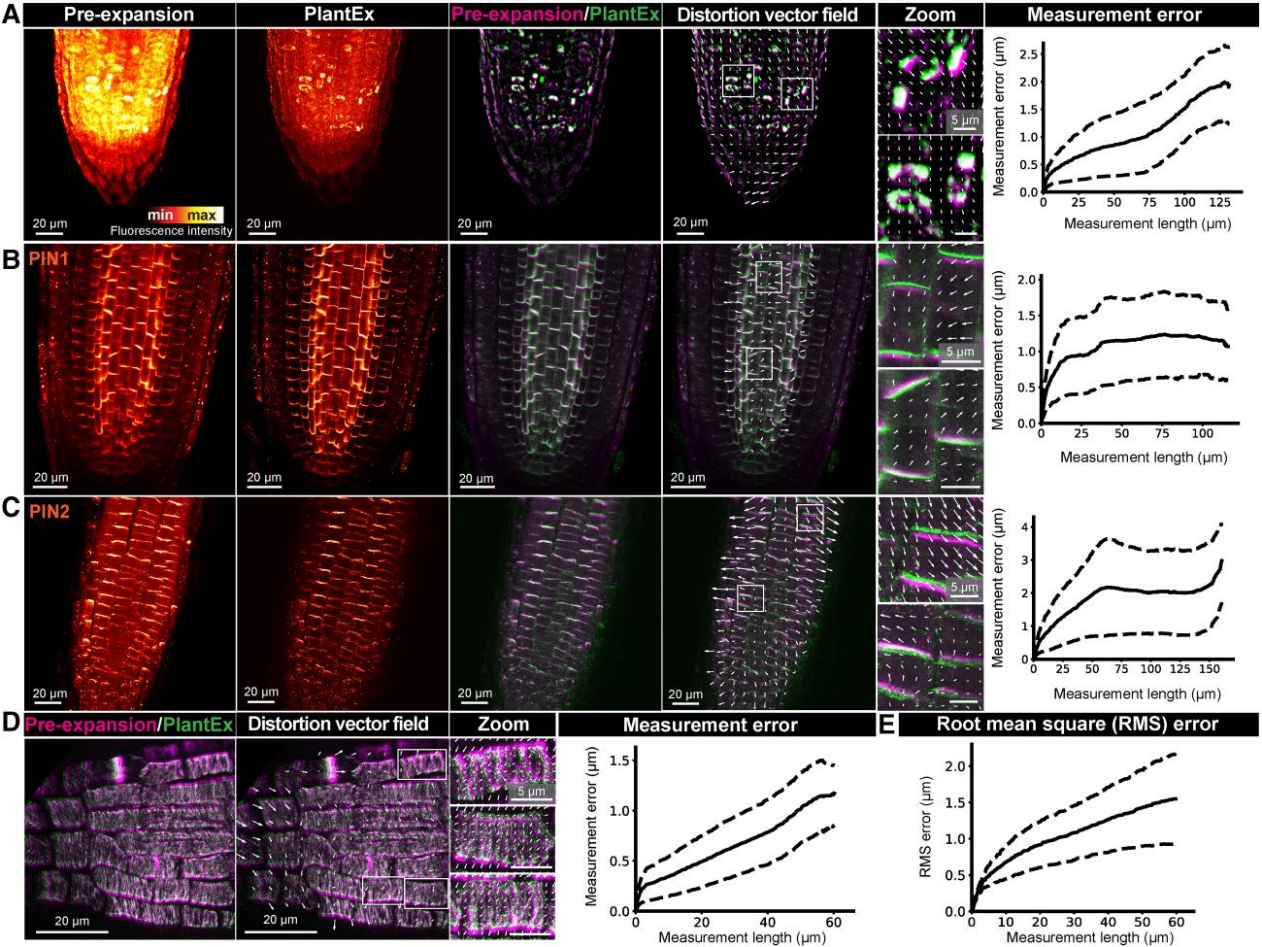
Characterization of distortions. **A)** Left to right: Maximum intensity projections of confocal overview image stacks of the same tubulin-labeled *A. thaliana* root tip before and after expansion (imaged with a low-NA objective lens). Overlay of scaled and aligned pre-expansion and PlantEx images and distortion vector field with zoomed views characterizing displacements between pre- and post-expansion images. For display purposes, the length of distortion vectors was scaled up 2.5-fold in the overview images. To focus distortion analysis on distinct image features, homogeneous components of the signal were removed (for details see the “Methods” and for image alignment see Supplementary Fig. S2). Plot of distortions between the pre- and post-expansion images as a function of measurement distance for the same sample. Numerical values for distances and distortions refer to pre-expansion scale throughout. Solid and dashed lines refer to mean and standard deviation, respectively. Scale bars: 20 µm (82 µm physical scale in the expanded sample, exF = 4.1). Scale bars, zoomed views: 5 µm. **B** and **C)** Similar measurements for PIN1 and PIN2 immuno-labeled roots, outlining cell membranes (pre-expansion imaging with 40×, NA1.2 objective lens, PlantEx imaging with 20×, NA0.8 objective). Scale bars: 20 µm (74 µm (PIN1) and 78 µm (PIN2) physical scale in the expanded samples, exF = 3.7 and 3.9, respectively). Scale bars, zoomed views: 5 µm. **D)** Overlay of pre- and post-expansion images, distortion vector fields, and distortions as a function of measurement length for the measurement in Fig. 1D. Scale bars: 20 µm (74 µm physical scale in the expanded sample, exF = 3.7). Scale bars, zoomed views: 5 µm. **E)** Distortions as a function of measurement distance, quantified as RMS error across the *n* = 4 samples in panels **A–D)** (see Methods). Measurement error showed greater variability and apparent drops at larger measurement lengths for individual measurements in panels **A–D)** due to bias from sampling distortion vector fields at the outermost regions of the applied masks. The RMS error calculated across multiple measurements in panel **E)** shows a smooth rise. Solid and dashed lines refer to mean and standard deviation, respectively.

To determine the fidelity at which cellular structures were expanded with PlantEx, we evaluated the magnitude of expansion-induced distortions. We aligned rigidly scaled pre-expansion and PlantEx images of root samples (Supplementary Fig. S2) and calculated displacement vector fields as well as the magnitude of distortions as a function of measurement length (Fig. 2). Overlaying overview images of the same tubulin-labeled hydrogel-embedded root taken before and after expansion (Fig. 2A) indicated that the expansion procedure maintained overall architecture. We next immunostained for the cell surface markers PIN-FORMED1 (PIN1) and PIN-FORMED2 (PIN2) auxin transporters located at the cell membrane, expressed in deep root meristem layers and in the outer layers of cortex and epidermis, respectively (Adamowski and Friml 2015; Omelyanchuk et al. 2016)—to evaluate expansion-induced distortions of cell outlines (Fig. 2B and C). Here, super-resolution imaging in deep layers of the root was facilitated by optical clearing mediated by hydrogel embedding and expansion, which homogenized refractive index and adjusted it to that of water, and suppressed fluorescence background. We further analyzed distortions at the tissue level (Fig. 2D) in the high-resolution data from the tubulin-labeled root in Fig. 1D. Taken together, we observed root mean square (RMS) displacements of a few percent of the measurement length (≲5%) between corresponding pre- and post-expansion images across these measurements (Fig. 2E), similar to previous work on mammalian tissue (Sun et al. 2021; Klimas et al. 2023). This demonstrated that expansion of plant tissues was possible at high fidelity with expansion factors equaling other sample types for the chosen gel chemistry. Enzymatic cell-wall digestion loosens the rigid polysaccharide structure while maintaining the integrity of plant architecture.

### Dual-channel PlantEx

**Figure 3.**
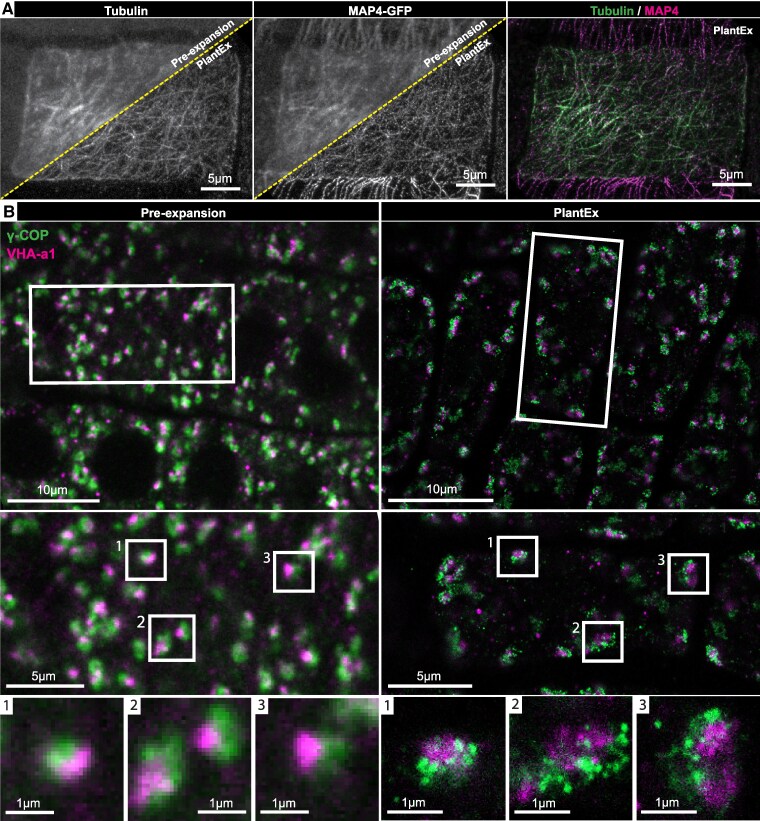
Dual-color PlantEx. **A)** Dual-color PlantEx measurement of tubulin and MAP4 in root tissue of an *A. thaliana* line expressing a MAP4-GFP fusion protein. Tubulin and GFP were visualized via immunolabeling. Left: Confocal PlantEx image of tubulin in a root cell, with the corresponding pre-expansion confocal image in the upper left corner. Middle: MAP4 channel. Right: Overlay of tubulin and MAP4 signal. In the specimen imaged here, intensity of pre-expansion immunolabeling was variable between cells (Supplementary Fig. S4). Scale bar: 5 µm. Maximum intensity projections covering approximately equal ranges in *z*-direction. **B)** Dual-color PlantEx measurements of COPI-coated vesicles and TGN. Left: Pre-expansion confocal images of lateral root cap tissue immunostained for GFP expressed as fusion protein with a TGN marker (vacuolar proton ATPase subunit a isoform 1, VHA-a1, magenta) and for the γ-subunit of COPI-coated vesicles (γ-COP, sec21, green). Cell boundaries can be discerned as regions devoid of stained structures. Right: Confocal image of a similar region in a different sample after applying PlantEx. Resolution and signal-to-background ratio were increased. Scale bars: 10 µm (39 µm physical scale in the expanded sample, expansion factor exF = 3.9). Center, bottom: Magnified views of regions indicated by boxes at cellular and subcellular scales. Examples with roughly corresponding structures were selected from pre-expansion and PlantEx images. Color maps are linear with saturation of brightest pixels. Scale bars: 5 µm (center) and 1 µm (bottom).

ExM does not require specific fluorophore properties or fluorophore state control and thus offers a facile route to multicolor super-resolution imaging. We sought to adapt PlantEx for multicolor imaging to further its applications for biological studies exploring nanoscale spatial relationships (Fig. 3). We first evaluated the spatial arrangement of microtubule-associated protein 4 (MAP4) and microtubules at extended resolution (Fig. 3A), using an established transgenic line (Marc et al. 1998) expressing a MAP4-green fluorescent protein (GFP) fusion protein. We used GFP as a tag, visualized it with immunolabeling, and combined it with immunolabeling for tubulin. As expected, MAP4 localized to microtubules (Marc et al. 1998), while we also observed variability in labeling brightness between cells in this measurement (Supplementary Fig. S4), likely due to differences in antibody access to cells in the immunolabeling performed before hydrogel embedding and expansion. This notion is supported by comparison with live confocal imaging of the same MAP4-GFP line (Supplementary Fig. S5), detecting GFP fluorescence directly rather than using antibody labeling. Live imaging of GFP-labeled roots provided a bright, homogeneous signal, albeit at lower resolution than with PlantEx.

We next extended the range of molecular targets and cellular structures by addressing the relationship between components of the Golgi apparatus (GA) and vesicle trafficking machinery (Neumann et al. 2003) (Fig. 3B). The plant GA consists of discontinuous entities dispersed throughout the cytoplasm. COPI-coated vesicles mediate cargo trafficking within the GA and retrograde Golgi-endoplasmic reticulum trafficking, and are indispensable for plant growth and development (Donohoe et al. 2007; Ahn et al. 2015). To elucidate the spatial arrangements of COPI-coated vesicles and the trans-Golgi network (TGN), part of the GA that relays vesicles to various cellular targets, we used an established *A. thaliana* transgenic line (Dettmer et al. 2006) where the TGN was highlighted by expression of GFP fused to the TGN-resident H^+^-ATPase VACUOLAR PROTON ATPASE SUBUNIT A ISOFORM 1 (VHA-a1). We detected both the GFP-tag and endogenous Sec21, the γ-subunit of the coatomer in COPI-coated vesicles (γ-COP), via immunostaining. Without expansion, using a high-NA objective lens for confocal imaging, these markers were often overlapping. In contrast, in PlantEx, the arrangement of the individual COPI-coated vesicles around the TGN perimeter was readily discernible (Supplementary Videos S1 and S2). While suggested in some locations in the pre-expansion imaging volume, this spatial relationship was robustly evident throughout most of the expansion imaging volume. This agrees with previous observations of COPI-vesicles budding from and trafficking between Golgi cisternae (Donohoe et al. 2007). Also here, the signal from immunolabeled cellular structures stood out more clearly against the cellular background in PlantEx. With dual-channel imaging, PlantEx thus facilitated detailed studies of cellular organization and relative molecular arrangements in plant samples.

### Combining PlantEx with existing super-resolution techniques

As an ExM technique, PlantEx increases effective resolution by means of a dedicated sample preparation procedure. The optical imaging modality, on the other hand, can be chosen according to measurement requirements. This strategy has been followed in diverse contexts for expanded hydrogels, including simple widefield imaging, modalities offering optical sectioning like confocal or light-sheet microscopy, and advanced imaging techniques, such as lattice light-sheet microscopy (Gao et al. 2019). To enhance effective resolution beyond the increase offered by the expansion factor, ExM has been combined with optical super-resolution imaging according to the specific strengths of individual methods for particular imaging tasks. Examples include single-molecule ((d)STORM) (Xu et al. 2019; Zwettler et al. 2020), STED (Gao et al. 2018; Gambarotto et al. 2019), SIM (Cahoon et al. 2017; Halpern et al. 2017), and fluctuation-based super-resolution imaging (Shaib et al. 2024), reaching further into the nanoscale regime than expansion alone.

**Figure 4.**
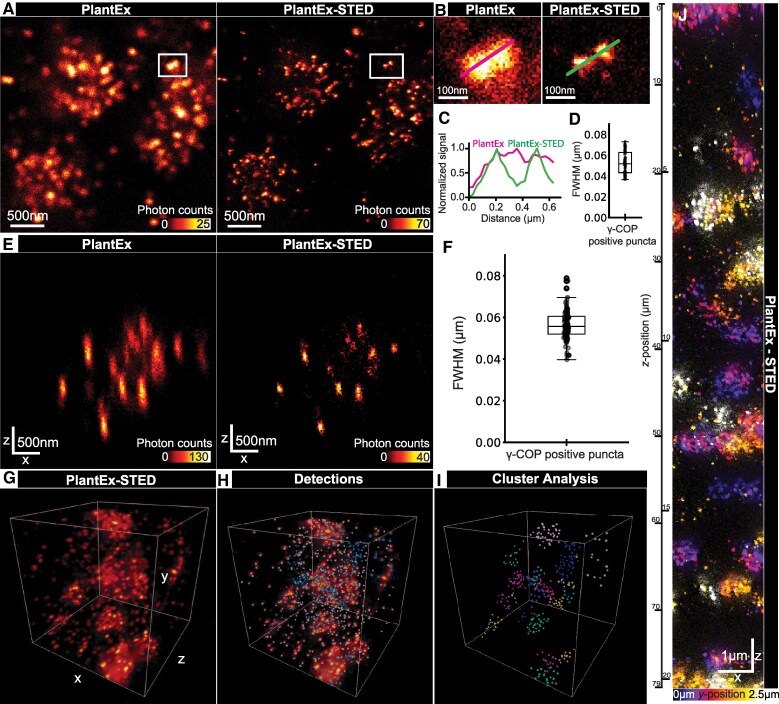
PlantEx with STED microscopy readout. **A)** Left: COPI-coated vesicles in an expanded *A. thaliana* whole-mount root sample labeled for γ-subunit of COPI-coated vesicles (γ-COP, sec21) (PlantEx), imaged with confocal microscopy. Right: Same region imaged with STED microscopy (PlantEx-STED), using an *xy*-STED light pattern (“doughnut” pattern, lateral resolution increase). Scale bar: 500 nm (1.95 µm physical scale in the expanded sample). Numbers in intensity lookup bar represent single-photon detection events. Color map is saturated at the brightest pixels (white). Images representative of STED experiments in *n* = 4 specimens. **B)** Magnified views as indicated by boxed regions in **A)**. Lines indicate positions of intensity profiles. **C)** Corresponding line intensity profiles. Distance scale refers to original tissue size. **D)** FWHM of 2D-Gaussian fits to γ-COP (sec21) positive puncta in PlantEx-STED with lateral resolution enhancement (median value: 52 nm, lower and upper quartiles: 43 and 63 nm, 74 puncta across *n* = 3 specimens). Box plot center line, median; box limits, upper and lower quartiles; whiskers, 1.5× interquartile range; points, individual data points. **E)** Left: Axial confocal scan (*xz*-direction) in a PlantEx sample with the same labeling. Right: Axial scan of the same region with STED light pattern (“*z*-STED pattern”) increasing resolution predominantly along the optical axis (*z*-direction). Scale bar: 500 nm (1.95 µm physical scale in the expanded sample). **F)** FWHM of 3D-Gaussian fits to γ-COP positive puncta in volumetric PlantEx-STED with *z*-STED pattern (median: 56 nm, lower and upper quartiles: 52 and 61 nm, 112 puncta across *n* = 3 specimens). Box plot center line, median; box limits, upper and lower quartiles; whiskers, 1.5× interquartile range; points, individual data points. **G)** 3D-rendering of PlantEx-STED imaging volume with labeling for COPI-coated vesicles (*γ*-COP) imaged at near-isotropic resolution (*z*-STED pattern). Fly-through: Supplementary Video S3. Imaging volume: 3.8 × 3.8 × 3.8 µm^3^ (15 × 15 × 15 µm^3^ physical scale in the expanded sample). **H)** Visualization of *γ*-COP positive puncta, detected as local intensity maxima in the same volume. **I)** Color coding after applying a clustering algorithm. Puncta not assigned to clusters are indicated in gray (Supplementary Video S7). **J)** PlantEx-STED imaging with extended axial range. COPI-coated vesicles imaged at near-isotropic resolution (*z*-STED pattern) in a tissue column of 79 µm imaging range in *z*-direction, corresponding to 20.25 µm in native tissue. Side view (*xz*-direction), the third dimension (*y*) is color-coded. Scale bar: 1 µm (3.9 µm physical scale in the expanded sample). Representative of volumetric STED imaging in *n* = 3 samples.

In our case, we were interested in further increasing the resolution of PlantEx in volumetric imaging to better visualize COPI-coated vesicles, as PlantEx with diffraction-limited confocal readout failed to resolve individual COPI-coated vesicles in densely packed regions of the cell (Fig. 4A and B). For this, combining PlantEx with the super-resolution imaging technique STED (Hell and Wichmann 1994; Klar et al. 2000; Jahr et al. 2020) microscopy was a convenient choice (Fig. 4), as (point-scanning) STED microscopy increases resolution by simply applying an additional light pattern to switch off fluorophores in the periphery of the diffraction-limited excitation volume. Paired with confocal detection for optical sectioning, this facilitates imaging in thick specimens. In terms of modifications to sample preparation, STED simply requires using STED-compatible fluorophores for labeling. STED can be performed in pure water rather than specialized buffers, which are e.g. necessary in (d)STORM to ensure fluorophore blinking. This simplifies imaging of expanded hydrogels, where use of ion-containing buffers would typically require an additional (nonionic) stabilizing hydrogel to avoid shrinkage of the expandable hydrogel. While STED microscopy has been applied to plants (Kleine-Vehn et al. 2011; Sims et al. 2021), it is usually difficult to perform in thick and opaque samples such as plant roots and thus often fails to produce equivalent performance as in other biological samples (Supplementary Fig. S6), with STED performance being sensitive to aberrations and scattering. However, as PlantEx provides optical clearing and refractive index matching, we reasoned that it would facilitate STED readout. Using a water immersion objective lens, we applied STED microscopy to expanded hydrogels (Gao et al. 2018; Gambarotto et al. 2019) of *Arabidopsis* seedlings immunolabeled for COPI-coated vesicles with a high-performance STED dye compatible with radical-based hydrogel polymerization (Fig. 4). This PlantEX-STED modality allowed us to capitalize on the combined resolution improvements offered by the individual PlantEx and STED methodologies. Dialing in STED resolution increases in the focal plane (*xy*-direction) in addition to PlantEx, resolved individual vesicles that coalesced with conventional confocal readout (Fig. 4A–C). The apparent diameter of COPI puncta in such PlantEX-STED images (Fig. 4D and Supplementary Fig. S7) was 52 nm (median full-width-at-half-maximum (FWHM), native tissue scale, lower and upper quartiles: 43 and 63 nm, 74 vesicles recorded across *n* = 3 specimens). This may be taken as a proxy for the effectively achieved resolution in this dual super-resolution mode, with the caveat that COPI-coated vesicles have a size of tens of nm (∼45 nm) themselves (Pimpl et al. 2000; Donohoe et al. 2007). Similarly, irrespective of the applied super-resolution readout technology, immunolabeling for an organelle protein component does not necessarily trace out its nanoscale shape due to a potentially limited coverage of the vesicle outline by the finite number of protein targets present, finite epitope coverage, and displacement of fluorophores from the target structure due to the non-negligible size of antibodies used for labeling. Individual antibodies measure ∼10 nm in size and primary and secondary antibodies may be spatially oriented in arbitrary directions in the immune labeling. Accordingly, such “linkage error” between biological target and fluorophore may increase the apparent size of COPI-coated vesicles by up to a few tens of nanometer, which is non-negligible compared to the actual organelle size, and affect measured values.

Given the anisotropic shape of the confocal point-spread function, we expected that the pivotal factor for discerning vesicles in 3D would be increasing resolution in the axial (*z-*)direction. We thus applied a STED light pattern for resolution increase predominantly in the *z*-direction (*z*-STED, Fig. 4E). Dialing in near-isotropic diffraction-unlimited resolution and employing reduction of state transition cycles (RESCue)-STED (Staudt et al. 2011) to mitigate photobleaching allowed volumetric PlantEx-STED imaging (Fig. 4G and Supplementary Fig. S8, Videos S3–S6). This was compatible with the detection of COPI-coated vesicles by a straightforward peak-finding algorithm, whereas they would largely coalesce in conventional diffraction-limited images or PlantEx alone. Automated detection allowed for visualization of their 3D spatial arrangement in tissue and we used a clustering algorithm to color code detections and highlight regions of high vesicle abundance (Fig. 4H and I and Supplementary Fig. S8, Videos S7–S10). We also quantified the apparent size of the COPI puncta in volumetric, near-isotropically resolved PlantEx-STED datasets with 3D-Gaussian fits, resulting in a median FWHM of 56 nm (native tissue scale, lower and upper quartiles: 52 and 61 nm, 112 vesicles recorded across *n* = 3 specimens) (Fig. 4F and Supplementary Fig. S7).

In tissue imaging, it is typically challenging to achieve super-resolution over an extended axial range, due to depth-dependent aberrations (e.g. spherical), scattering, and photobleaching. Hydrogel-based refractive index homogenization in combination with a high-NA water immersion objective lens largely solves these issues. Together with RESCue STED, this allowed us to image the 3D-distribution of COPI-coated vesicles in a hydrogel column of ∼80 µm axial extent (Fig. 4J), corresponding to ∼20 µm in the native tissue, within a single measurement. Together, this demonstrated that PlantEx can be advantageously combined with advanced optical readout modalities, as exemplified here with STED microscopy to improve overall achievable resolution.

### Comprehensive visualization of tissue architecture in PlantEx

**Figure 5.**
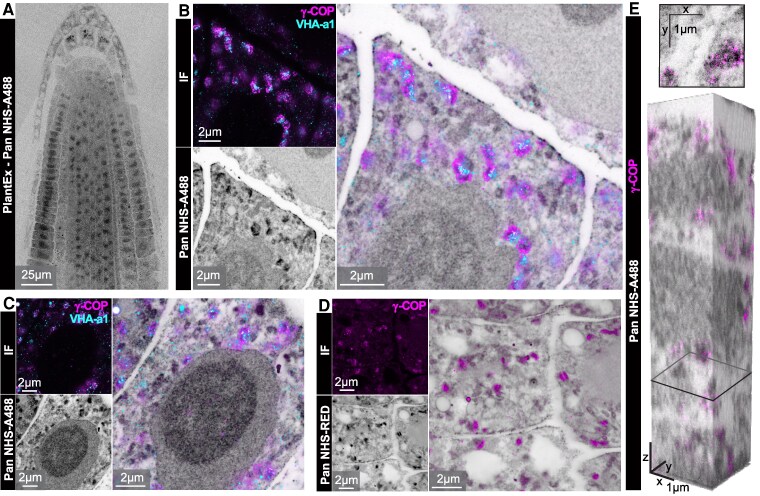
PlantEx with comprehensive visualization of tissue architecture. **A)** Spinning-disk confocal overview image of expanded *A. thaliana* whole-mount root sample with “pan”-labeling using an NHS-modified fluorophore (grayscale, NHS-A488: NHS-Alexa 488) to map protein density. Grayscale color map is inverted (black represents high protein density, noninverted images: Supplementary Fig. S9). Scale bar: 25 µm (97.5 µm physical scale in the expanded sample). **B)** TGN and COPI-coated vesicles in cellular context. Top left: PlantEx-STED image of vacuolar proton ATPase subunit a isoform 1 (VHA-a1, cyan, visualized by immunofluorescence labeling for GFP-tag, *xy*-STED) and *γ*-COP (immunolabeling for sec21, magenta, *xy*-STED) in whole-mount *A. thaliana* root PlantEx specimen. Bottom left: Same region visualizing cellular architecture with pan-protein label (confocal, grayscale, color map inverted, NHS-A488). High intensity in pan channel at TGN/vesicle locations partially reflect increased protein density from antibodies applied before NHS-fluorophore labeling. Right: Overlay. Scale bars: 2 µm (7.8 µm physical scale in the expanded sample). **C)** Similar measurement in different region (*γ*-COP with *xy*-STED, VHA-a1 confocal). Scale bars: 2 µm. **D)** PlantEx of cellular architecture in whole-mount *A. thaliana* root sample with pan-protein labeling, read out with STED microscopy (grayscale, *xy*-STED, NHS-RED: NHS-Star Red, colormap inverted), with COPI-coated vesicles (*γ*-COP, magenta, confocal). Scale bars: 2 µm. For the overlay, background counts in the immunolabeling channel were subtracted for visual clarity. **E)** Volumetric PlantEx-STED imaging with cellular context. COPI-coated vesicles (*γ*-COP, magenta, *z*-STED) in cellular context of pan-protein labeling in *A. thaliana* PlantEx root sample (confocal, grayscale, NHS-A488, colormap inverted). Top: Cross section at the position indicated in the bottom panel. Bottom: 3D-rendering of volume (2.6 × 2.6 × 12.8 µm^3^ native scale). Scale bars: 1 µm. Representative of pan-protein labeling in *n* = 3 samples.

We were next interested in providing a means to comprehensively visualize tissue architecture at super-resolved subcellular scale with PlantEx. We hence applied fluorophores bearing an N-hydroxysuccinimide (NHS) ester to PlantEx samples (Fig. 5 and Supplementary Fig. S9), inspired by visualization of cell and tissue architecture in mammalian cells and tissues (Mao et al. 2020; M'Saad and Bewersdorf 2020; Damstra et al. 2022; M'Saad et al. 2022) through indiscriminate (“pan”) protein-density staining. These covalently reacted with (primary) amines abundant on cellular proteins and thus mapped cellular structure comprehensively. Such labeling clearly outlined cell shapes and we used it to place specific molecular signals into the context of subcellular architecture at resolution 4-fold increased by PlantEx (Fig. 5B–E). For this, we imaged the structural channel with confocal microscopy and visualized the TGN and COPI-coated vesicles at further augmented resolution with STED microscopy (Fig. 5B and C). Next, we applied NHS-ester modified STED-compatible fluorophores to expanded root samples, thus enabling further resolution increase with STED also in the structural channel (Fig. 5D). Protein-density labeling in PlantEx also provided cellular context in volumetric datasets, when we imaged COPI-coated vesicles in PlantEx-STED mode at near-isotropic resolution (using a *z*-STED pattern) and read out the pan channel with confocal microscopy (Fig. 5E).

We also found that indiscriminate protein labeling was practically useful to locate the otherwise transparent PlantEx samples in 3D within the hydrogel for imaging, also when using a low-NA objective lens with moderate photon collection efficiency. More importantly, comprehensive structural labeling provided a general and straightforward way of assessing tissue architecture and preservation in expanded plant samples (Fig. 5A). As plant seedlings are delicate specimens, we found this a convenient way to rapidly gauge whether a particular specimen had been damaged during manual handling for fixation, labeling, or hydrogel embedding, and it allowed for an instantaneous assessment of homogeneity of expansion independent of immunolabeling. Taken together, pan-protein labeling substantially extends the information content in PlantEx imaging.

## Discussion

Here, we developed ExM for plant tissues, using the intact *A. thaliana* root as a model system, thus overcoming the notoriously unfavorable optical properties of plants for super-resolution imaging. PlantEx offers a 4-fold increase in effective resolution over the chosen readout modality in all 3 spatial directions for multiple color channels, and preserves *planta* structures with high fidelity using several probes. This provides effective super-resolution also with widely available conventional diffraction-limited microscopes, such as confocal systems. It thus opens up the prospect of nanoscopic imaging studies for plant biology laboratories not specialized in, or lacking access to, classical super-resolution imaging. We showcased how PlantEx may facilitate biological discovery, visualizing multiple molecular targets, including cytoskeletal organization and nanoscale arrangement of organelles. Notably, PlantEx is compatible with existing well-characterized FP-expressing marker lines by immunolabeling for the FP tag. We also demonstrated how PlantEx can be further enhanced by choosing advanced optical imaging modalities according to the application requirements. We combined it with volumetric STED super-resolution imaging in the intact tissue to resolve individual COPI-coated vesicles in dense clusters associated with the GA. We also used this system to highlight that the improved resolution and signal-to-noise ratio offered by PlantEx facilitate analysis of biological specimens with automated approaches.

As in any expansion technology, resolution increase in PlantEx is equivalent to the expansion factor (4×, ∼65 nm lateral resolution at 490 nm excitation with confocal readout using an NA = 1.2 objective lens). Hydrogel embedding and expansion also optically clear the sample, by removing cellular constituents such as lipids, cell-wall components and chlorophyll, and by homogenizing and adjusting the refractive index to that of water. Endogenous fluorophores likely fail to be anchored or may be destroyed during polymerization and homogenization. The rapid nature of PlantEx sample preparation offers advantages over existing time-consuming plant clearing methods (Palmer et al. 2015). Clearing and index matching benefit optically demanding readout modalities. For instance, the quality of the intensity minimum of a *z*-STED pattern for axial resolution increase (Klar et al. 2000) is highly sensitive to spherical aberrations and scattering, posing a challenge in native plant samples, whereas this is largely abolished when using water immersion objectives with PlantEx hydrogels, even when imaging whole-mount samples as we did here. It stands to reason that PlantEx can advantageously be combined with further optical imaging modalities depending on measurement requirements, such as light-sheet microscopy.

The currently achieved 4-fold resolution increase compares favorably against the more moderate gains in resolution with Airyscan or linear SIM, while these modalities bear the advantage of being also applicable to living samples. Also, RESOLFT (Schnorrenberg et al. 2020) is applicable to living specimens but requires specialized equipment and engineering of transgenic lines expressing FPs with specific (photoswitching) properties. Optical super-resolution techniques have been applied to plant samples to achieve high resolution, down to e.g. 40 nm in the case of STORM (Dong et al. 2015). However, optical aberrations, scattering, and fluorescence background do render these challenging for tissue imaging, especially for accessing deeper portions of the tissue. Through combination with STED readout, it was straightforward to achieve resolution increase in PlantEX beyond the 4-fold expansion. Incorporating alternative hydrogel chemistries (Chang et al. 2017; Truckenbrodt et al. 2018; Damstra et al. 2022; Klimas et al. 2023) may in the future provide a route toward further resolution improvements using standard microscopes.

Beyond the obvious restriction of ExM to fixed specimens, a series of considerations apply when adopting the technology. Common to all super-resolution approaches, the technology visualizes fluorophores rather than the actual biological target molecules. Hence, biological structures need to be sampled by fluorophores at sufficient labeling density for adequate visualization. While the discrete spatial sampling by fluorophores is typically concealed at diffraction-limited resolution, it often becomes apparent at the tens of nm resolutions achieved with PlantEx and even more so when resolution is further increased in PlantEx-STED. The overall imaging outcome thus crucially depends on the quality of the initial antibody staining and we showed how an improved immunolabeling protocol enhanced visualization of microtubules in PlantEx. In our protocol, hydrogel embedding and expansion ensued after immunolabeling. It is hence only applicable to targets where labeling can be performed in the native tissue. Despite the initial, mild cell-wall digestion step, antibody penetration into the tissue may vary across specimens and may result in labeling restricted to superficial cell layers. In addition, with pre-expansion immunolabeling, antibody access or labeling intensity may also vary between individual cells (see data in Fig. 3A and Supplementary Fig. S4), which is a common observation for plant immunolabeling protocols. It is thus prudent to evaluate labeling quality and brightness before expansion, as signal dilution and fluorophore loss during expansion will reduce labeling intensity. Common to all expansion approaches with pre-expansion immunolabeling, only labels retained in the hydrogel can be detected post-expansion. Well-known tradeoffs exist in protein-retention ExM between the degree of mechanical homogenization and retention of fluorophore-bearing antibody fragments after proteolytic treatment (Truckenbrodt et al. 2019). Stronger digestion increases fluorophore loss and we point out that certain fluorophores, notably cyanine dyes, are not compatible with the radical chemistry during hydrogel formation. Specific to PlantEx, the plant-specific cell-wall digestion cocktail also displays proteolytic activity, which should be taken into account when optimizing mechanical homogenization including proteinase K digestion. Specific optimization may indeed be required, as the cocktail includes natural extracts whose activity may vary between batches. Another caveat common to approaches involving (pre-expansion) immunolabeling is a displacement of fluorophores from target structures, as fluorescence imaging visualizes fluorophores rather than the molecules or structures of interest themselves. Such linkage error may reach ∼20 nm with the secondary immunostainings applied here, as antibodies are large molecules of ∼150 kDa and ∼10 nm size. Furthermore, the spatial arrangement and orientation of antibodies with respect to the target epitopes is not known. A low copy number of target proteins on individual cellular organelles or incomplete epitope coverage may impede outlining the precise shape of an organelle. Consequently, measurements based on antibody labeling of vesicle proteins are useful to put bounds on resolution rather than for determining the precise organelle size. There is potential for improving these various factors by development of approaches using smaller probes (e.g. nanobodies), post-expansion labeling (Ku et al. 2016), or optimized signal retention strategies (Wen et al. 2020). For quantitative biological measurements, validation strategies for each cellular target must be adapted to the biological question posed, including homogeneity of expansion of the target structure.

Beyond the obvious benefit of visualizing tissue architecture comprehensively and providing super-resolved subcellular context, we found protein-density mapping (“pan-protein” labeling) a useful way to assess structural preservation for individual samples and to locate the plant root in the expanded hydrogel, as the cleared seedlings produce hardly any nonfluorescent contrast and immunolabelings can be faint. Visualization of specific molecules together with comprehensive structural representation of the tissue's architecture can also be achieved with correlative light/electron microscopy (CLEM). Here, light microscopy provides information on the location of specific molecules while EM provides a comprehensive structural representation at high resolution. Recent advances in the plant imaging field have e.g. allowed pairing single-molecule super-resolution imaging with transmission electron microscopy (TEM) on the same thin (150 nm), resin-embedded sections of *A. thaliana* roots (Franek et al. 2024). Both PlantEX and CLEM methods are designed for fixed specimens, such that they cannot access the dynamics of the living system, and chemical fixation and subsequent sample preparation procedures cause alterations from the native living state. Advantages of PlantEx include straightforward volumetric imaging, owing to the optical sectioning capability of various widely available optical microscopy modalities (e.g. confocal). Furthermore, the molecular information and structural context are obtained with the same microscope, obviating the necessity for image alignment across modalities. Serial sectioning TEM and other EM technologies, such as focused ion beam milling–scanning electron microscopy enable volumetric EM imaging (Collinson et al. 2023), and specialized protocols have been developed for plants (Wickramanayake and Czymmek 2023). However, correlation with light microscopy requires expertise in 2 domains and equipment that may not be as readily available to researchers, while a clear advantage of electron microscopy is its unrivalled detail in the structural images.

Our protocol was developed using the *A. thaliana* root organ, thus enabling cell biological and developmental studies in this important model system. We expect that this will also spark adaptation to other species. This will require tuning of the labeling and mechanical homogenization procedures, as different species are expected to feature different molecular and mechanical characteristics, in particular regarding cell-wall composition. Similarly, expansion of different organs will require dedicated protocols. Both aspects are akin to the application of ExM to animal samples, where a wide variety of protocols have been developed to cater for differences according to species or tissue imaged. We note that concurrently and independently of our work, Grison et al. (2024) have developed a similar protocol for expansion of whole-mount *A. thaliana* root samples. This provides corroboration of the overall approach and, based on the differences in specific aspects, pointers to where future adaptations may be possible.

Overall, PlantEx enables accessible super-resolution imaging to facilitate novel cell biological studies that examine subcellular entities and their mutual spatial relationships. Furthermore, PlantEx will enable the decoding of complex 3D topologies of cellular organelles and the spatial interplay with interaction partners, giving access to cellular nanoscale architecture in a molecularly informed way.

## Materials and methods

### Plant material and growth conditions

Thale cress (*A. thaliana*) ecotype Col-0 was used for tubulin staining. For imaging of COPI-coated vesicles and TGN, a VHA-a1-GFP-expressing line (Dettmer et al. 2006) with a Col-0 background was used. For visualization of MAP4, a MAP4-GFP line (Marc et al. 1998) with a Col-0 background was used. Seeds were surface-sterilized with chlorine gas (generated from 39 ml of 12% sodium hypochlorite-based bleach, 61 ml of H_2_O, and 4.5 ml of 37% HCl) overnight or with a 15 min 70% ethanol wash followed by a 15 min 100% ethanol wash and then air-dried under sterile conditions. Following a 48–72 h 4 °C stratification, seedlings were germinated and grown on ½ Murashige–Skoog (MS) medium (1% D(+)-Saccharose (Sigma 84100), 0.23% MS powder (Duchefa M0221.0050), 0.05% MES (2-(N-morpholino)-ethanesulfonic acid) (Duchefa M1503.0100), 0.8% Agar (Duchefa P1001.1000), with pH set to 5.6 with KOH prior to autoclaving) for 3–5 days in a growth room at 21 °C and long-day conditions (16 h light, 8 h dark, LED Philips GreenPower Production module DR/B/FR; PPFD [µmol/m²/s]: 139.9, PFD-B: 16.35, PFD-G: 0.14, PFD-R: 121.66, and PFD-FR: 7.76).

### Fixation and immunostaining

Fixation was done with paraformaldehyde and immunostaining was either performed manually or with a pipetting robot. Labeling protocols included a mild first cell-wall digestion step to allow for antibody access but maintain tissue integrity. Thorough cell-wall digestion for PlantEx was performed after anchoring and hydrogel polymerization.

#### Tubulin labeling for ExM

For tubulin staining, we followed a previously published fixation and labeling protocol, which is described in full detail in Pasternak et al. (2015). In brief, about a dozen of seedlings aged 3–4 days were transferred from the agar plate and fixed in a 12 well plate with 2% paraformaldehyde in 1× microtubule stabilization buffer (MTSB) supplemented with 0.1% Triton X-100 (Sigma-Aldrich, T8787). 2× MTSB stock solution was: 15 g PIPES (piperazine-N,N′-bis(2-ethanesulfonic acid), Sigma-Aldrich, P1851)), 1.90 g EGTA (ethylene glycol-bis(*β*-aminoethyl ether)-N,N,N′,N′-tetraacetic acid, Sigma-Aldrich, E3889), 1.22 g MgSO_4_·7 H_2_O (Merck Chemicals, 230391), and 2.5 g KOH (Merck Chemicals, 105033) in 500 ml water, adjusted with KOH to pH 7.0. Samples were placed in a vacuum desiccator and suction was applied for 5 min, pressure brought back to ambient values, and suction applied again for 5 min to facilitate penetration of fixation solution. Fixation proceeded for additional 40 min at 37 °C and samples were washed for ∼10 min in 2 ml water and placed overnight in water at 4 °C. Next, seedlings were incubated for 10 min in 800 µl methanol at 65 °C, which solubilizes the plant cuticle and further components, such as chlorophyll. We then added 200 µl of Millipore water every 2 min until the final alcohol concentration reached ∼20% and washed seedlings twice for 5 min with water. Cell-wall digestion for antibody access was performed with a solution containing 0.2% driselase (Sigma-Aldrich, D8037) and 0.15% macerozyme R-10 (Yakult Pharmaceutical Industry Co., Ltd.) in 2 mm MES, pH 5.0 for 40 min at 37 °C. After washing for 4 min in MTSB, membranes were permeabilized with 3% IGEPAL CA-630 (Sigma-Aldrich, I8896), 10% DMSO (Sigma-Aldrich, W387520) in 1× MTSB for 20 min at 37 °C. After 4 washes for 3 min in 1× MTSB, samples were blocked in 1× MTSB supplemented with 2% bovine serum albumin (BSA, Sigma-Aldrich, A2153) for 30 min and incubated 1st with primary antibody (anti-Tubulin, Santa Cruz Biotechnology, SC-53030, monoclonal, IgG2a, 1:100, in blocking solution), washed 2 × 5 min in MTSB, and then with secondary antibody (Alexa Fluor 488, Goat anti-rat IgG (H + L) Cross-Adsorbed Secondary Antibody, Invitrogen, A 11006, 1:100, in blocking solution) for 2 h each and washed again 3 × 5 min in MTSB. If anchoring was not done on the same day, samples were stored in storage solution containing 90% Glycerol (VWR, ICNA0219399691), 10% PBS, DABCO (25 mg/ml, 1,4-Diazabicyclo[2.2.2]octane, Sigma-Aldrich, D27802), with pH adjusted to 9.

#### Optimized tubulin labeling

Manual fixation and immunolabeling were performed according to a previously published protocol (Vavrdová et al. 2019), with minor modifications. 3–4-days-old *A. thaliana* seedlings were transferred into a well plate and treated with 10 nm Taxol in water for 30 min and then were fixed in 1.5% formaldehyde, 0.1% glutaraldehyde with 0.01% Triton X-100 in MTSB for 1 h at room temperature. Samples were gently transferred onto a chambered slide and incubated at room temperature (RT) for 30 min in a cell-3 × wall digesting solution of 2.5% Cellulase Onozuka R10 (Yakult Pharmaceutical Industry Co.), 1% Macerozyme R10 (Yakult Pharmaceutical Industry Co.), 1% Meicelase (Desert Biologicals), 0.1% Pectolyase (Duchefa, P8004) in MTSB. Samples were washed 3 × with MTSB and 3 × with PBS pH7.4 for 5 min each at room temperature. Washing steps were performed with ∼100 µl volume. Since glutaraldehyde was used for fixation, the seedlings were then incubated for 15 min in 0.1% sodium borohydride in PBS. Following this aldehyde reduction step, the samples were permeabilized by 15 min incubation in 10% DMSO, 2% IGEPAL CA-630 (Sigma-Aldrich, I8896) and 0.01% Triton X-100 in PBS. Samples were then washed 3 times in PBS for 5 min each and were incubated overnight in a blocking solution consisting of 3% BSA (Sigma-Aldrich, A2153) and 0.5% BSA-c (polyacetylated BSA, Aurion) in PBS. Seedlings were 1st incubated overnight at RT in 100 µl primary antibody solution (anti-*α*-tubulin, mouse monoclonal, Sigma-Aldrich, T6074, 1:100 and anti-*β*-tubulin, mouse monoclonal, Sigma-Aldrich, T5293, 1:100) followed by 6 washes in PBS for 10 min each. Optionally, for MAP4-GFP detection, additional labeling was performed with rabbit anti-GFP (Agrisera, AS152987) and using rat anti-*α* tubulin for microtubules (anti-tubulin, Santa Cruz Biotechnology, SC-53030). The samples were blocked for 1 h at RT and then incubated in secondary antibody solution (Alexa Fluor 488, goat anti-mouse IgG (H + L) Highly Cross-Adsorbed Secondary Antibody, Invitrogen, A-11029, 1:300) overnight at 37 °C. For dual-color labeling with MAP4-GFP, Alexa Fluor 488 goat anti-rabbit (A11034, Thermo Fisher) and Alexa Fluor 633 goat anti-rat (A21094, Thermo Fisher) secondaries were used. The next day, samples were thoroughly washed in PBS (3 times, 5 min each) and mounted for inspection in a solution of 90% Glycerol (VWR, ICNA0219399691), 10% PBS, DABCO (25 mg/ml, Sigma-Aldrich, D27802), pH 9.

#### Robot-based labeling for PlantEx

We followed a previously published automated labeling protocol (Sauer et al. 2006). 3–4 days-old seedlings were fixed in 24 well-plates with 1 ml of 4% paraformaldehyde in PBS for 1 h in the vacuum desiccator with suction applied. Using an Intavis InSitu Pro robot, we incubated 3 times for 15 min in PBS + 0.1% Triton X-100 at RT, followed by 3 times 15 min in ddH_2_O + 0.1% Triton X-100. Initial cell-wall digestion was performed for 30 min with 2% driselase in PBS at 37 °C. Samples were washed 3 times for 15 min in PBS + 0.1% Triton X-100 at RT. Permeabilization was performed for 2 times 30 min with 10% DMSO and 3% Igepal CA-630 in PBS at RT, followed by 3 times washing for 15 min in PBS + 0.1% Triton X-100 at RT. Blocking was done for 1 h with 2% BSA in PBS. Samples were incubated for 4 h at 37 °C with 1 or 2 of the following primary antibodies in blocking solution as indicated for the respective measurements: anti-GFP from mouse (monoclonal, Sigma G6539, dilution 1:500, target: GFP); anti-sec21 from rabbit (raised against *A. thaliana*-derived sec21-peptide, Agrisera AS08327, dilution 1:500, target: sec21, the *γ*-subunit of COPI-coated vesicles (Movafeghi et al. 1999)); noncommercial custom antibodies raised in rabbit against PIN1 (Paciorek et al. 2005) or PIN2 (Abas et al. 2006). Samples were then washed 3 times for 15 min at RT in PBS + 0.1% Triton X-100. Incubation with secondary antibodies according to the host species of the applied primary antibodies was done in blocking solution at 37 °C for 4 h using anti-rabbit IgG from goat, highly cross-adsorbed, conjugated to Alexa Fluor 488 (Invitrogen A11034, dilution 1:100) and/or anti-mouse IgG from goat, conjugated to Alexa Fluor 594 (Abcam, 150116, dilution 1:100). In the pre-expansion images in Fig. 3B, a combination of 2 anti-GFP antibodies from mouse was used (anti-GFP from mouse, Sigma G6539, dilution 1:500, anti-GFP from mouse, Thermo Fisher, A11120, dilution 2 µg/ml), detected with anti-mouse IgG from goat, conjugated to Alexa Fluor 594 (Thermo Fisher, A11005, dilution 1:100). Sec 21 was labeled with anti-sec21 antibody as above, detected with anti-rabbit IgG from goat, conjugated to Star635P Abberior, ST635P-1002-500UG, dilution 1:100).

Samples were washed 3 times for 15 min in PBS + 0.1% Triton X-100 at RT and then 3 times 15 min in ddH_2_0 at RT. If anchoring was not done on the same day, samples were stored in storage solution.

#### Immunostaining for PlantEx-STED

We followed an identical protocol as in multicolor labeling above but used an anti-rabbit IgG from goat conjugated to Star635P (Abberior, Göttingen, Germany, dilution 1:100).

For immunolabeling prior to pan-protein labeling (see in the following sections), the following combinations with fluorophore-conjugated secondary antibodies were used: Pan-labeling with NHS-Alexa488 together with anti-rabbit IgG from goat conjugated to Star635P (Abberior, ST635P-1002-500UG, dilution 1:100) and anti-mouse IgG from goat, conjugated to Alexa Fluor 594 (Thermo Fisher, A11005, dilution 1:300); pan-labeling with NHS-STAR RED, together with anti-rabbit IgG from goat conjugated to Star580 (ST580-1002-500UG, Abberior, Göttingen, Germany, dilution 1:100) and anti-mouse IgG from goat conjugated to Alexa Fluor 488 (Invitrogen, A-11029, 1:300).

### Hydrogel embedding and expansion

Whole immunostained seedlings were transferred in PBS onto a large coverslip prepared with a hydrophobic pen (Invignome) to contain fluids. Typically, only 1 single seedling was placed per coverslip. Further mechanical manipulation or transfers of the seedling were avoided. Fluid was exchanged to anchoring solution (0.2 mg/ml Acryloyl-X, SE; Thermo Fisher Scientific, A-20770 in 1× PBS) and the sample was incubated overnight in a humid chamber at RT. For assembly of a gelation chamber similar to the ones previously described (Truckenbrodt et al. 2019), anchoring solution and the hydrophobic rim were removed and a chamber was built up by placing a strip of #1.5 cover glass on either side of the seedling and covering the assembly with a further 22 mm × 22 mm cover glass on top. The hydrogel monomer solution contained 8.625% (w/w) sodium acrylate (Sigma-Aldrich, 408220 or AK Scientific R624), 2.5% (w/w) acrylamide (Sigma-Aldrich, A9099), 0.15% (w/w) N,N'-methylenebisacrylamide (Sigma-Aldrich, M7279), and 2 m NaCl (Sigma-Aldrich, S7653) in PBS. Gelation of monomer solution was initiated by adding 0.2% w/w final concentration each from solutions containing 10% ammonium persulfate (Sigma-Aldrich, A3678) and 10% TEMED (Sigma-Aldrich, T7024) in ddH_2_O. Gelation chambers were filled with monomer solution and gels were allowed to polymerize for at least 3 h at 37 °C. After gelation, pre-expansion images were taken on a confocal microscope. For digestion of cell walls, gelation chambers were disassembled with a razorblade and gels were individually placed in wells of a 12-well plate. Digestion proceeded for 24 h at RT in the dark with slight shaking in 2 ml of a solution containing 2% driselase, 1.5% cellulase Onozuka R-10 (Yakult Pharmaceutical Industry Co.), 0.4% macerozyme R-10, 0.2% xylanase (Sigma-Aldrich, X2753) and 0.2% pectolyase Y-23 (Duchefa, P8004) in 1× PBS. Prior to application, the enzyme cocktail was allowed to sediment for 30 min and the supernatant was filter sterilized (0.22 µm filter) to remove debris. Gels were then washed twice in 2 ml of PBS for 15 mins each. Mechanical homogenization was performed in 2 ml of proteolysis solution containing proteinase K and Ca^2+^ ions (8 U/ml proteinase K (Sigma-Aldrich, P4850), 50 mm TRIS (AppliChem, A3452), 800 mm guanidine HCl (Sigma-Aldrich, G4505), 2 mm CaCl_2_ (Sigma-Aldrich, C5670), 0.5% (v/v) Triton X-100 (Sigma-Aldrich, 93426) in ddH_2_O, pH 8.0) overnight at 50 °C in the dark.

For expansion, gels were placed in 12 cm diameter dishes in the dark with an excess of ddH_2_O. Water was exchanged every 15 min until gels were fully expanded, typically after 3 fluid exchanges.

### Pan-protein staining

Hydrogels were incubated with NHS-ester-modified dye (20–40 µM in PBS, 2 ml volume) for 2 h at RT with gentle shaking. For confocal measurements, NHS-Alexa488 (APC-002-1, Jena Bioscience, Germany) was used. For STED imaging, NHS-STAR RED (STRED-0002-1MG, Abberior, Germany) was used. After labeling, samples were washed 3× for 15 min in PBS and expanded in ddH_2_O before imaging.

### Proteolysis assay

Protease activity was assessed with the EnzChek Protease Assay Kit (E6638, Thermo Fisher) according to the manufacturer's instructions. Concentrations of the PlantEx cell-wall digestion cocktail and proteinase K, buffers, incubation times, and temperatures were adjusted to the respective experimental steps in the PlantEx protocol. Fluorescence readout was performed with a spectrophotometer. For experiments involving protease inhibitor, 1 tablet of protease inhibitor (cOmplete, 11836153001, Sigma-Aldrich) was added to the PlantEx cell-wall digestion cocktail. Experiments involving enzymes targeting specific cell-wall components were done with *α*-amylase 10 U/ml, *α*-L-arabinofuranosidase 2.5 U/ml, *β*-mannanase 50 U/ml, cellulase 14 U/ml, pectate lyase 5 U/ml, and xyloglucanase 10 U/ml.

### Imaging

#### Sample mounting

Expanded gels were trimmed to contain the root and transferred to a home-made imaging chamber manufactured as previously described (Truckenbrodt et al. 2019).

#### Confocal microscopy

Pre-expansion and PlantEx images were acquired on Zeiss LSM-800 confocal microscopes equipped with plan-apochromat 10×/0.45 NA or 20×/0.8 NA air and plan-apochromat 40×/1.2 NA water immersion objectives. Excitation wavelengths were 488 and 561 nm. Confocal images were also acquired on an Abberior Instruments Expert Line STED microscope in confocal mode, using a 60×/1.2 NA water immersion objective lens.

#### Spinning disc confocal microscopy

An Andor Dragonfly microscope based on a Nikon Ti2E inverted microscope, with an Andor Zyla 4.2-megapixel sCMOS camera, was used for overview imaging, with continuous wave laser excitation at 488, 561, and 637 nm as appropriate. Overview images of single plant seedlings were acquired with a 20× air objective (Nikon CFI P-Apo 20× lambda/NA 0.45/WD 4.0 mm). Andor Fusion software was used for data acquisition.

#### STED microscopy

For PlantEx-STED measurements, expanded samples were imaged on an Abberior Instruments Expert Line STED microscope, with a 775 nm STED wavelength and ∼1 ns long STED pulses at a repetition rate of 40 mHz. Excitation wavelengths were ∼640 and ∼560 nm. Nanosecond time gating of fluorescence detection was used throughout STED measurements with a gating window of 8 ns duration. Samples were imaged with a 60×/1.2 NA water immersion objective. Stated power levels refer to the power at the front lens of the objective and bear an estimated uncertainty of 15%. STED power was distributed to the *xy*-“doughnut” pattern for resolution increase in the focal plane and the *z*-STED pattern for resolution increase along the optical axis according to the lateral/axial STED power ratios stated below with the imaging parameters. Where indicated, RESCue STED (Staudt et al. 2011) was applied with the parameters stated below, reducing light exposure and photobleaching. At each scan position, fluorescence photon flux was monitored and lasers were turned off in case fluorescence detection failed to reach a lower threshold or it reached an upper threshold, such that the signal could be extrapolated to the full pixel dwell time.

#### Live confocal imaging

The 5-day-old seedlings were mounted on a standard glass slide with a #1.5 rectangular 24 mm × 50 mm coverslip in ½MS medium without agar, and sealed with nail polish. Imaging was performed with a Zeiss LSM-800 microscope, using a 40×/1.2 NA water immersion objective.

### Imaging parameters

Figure 1D and E. Pre-expansion: objective lens 40×/1.2 NA; lateral pixel size 100 nm; total imaging *z*-range 60.75 µm; axial step size 750 nm; pixel dwell time 0.35 µs. PlantEx: objective lens 40×/1.2 NA; lateral pixel size 90 nm; total imaging *z*-range 42 µm; axial step size 750 nm; pixel dwell time 0.66 µs.

Figure 1G and H. Pre-expansion: objective lens 40×/1.2 NA; lateral pixel size 100 nm, total imaging *z*-range 21 µm, axial step size 1 µm; pixel dwell time 0.48 µs.

PlantEx: objective lens 20×/0.8 NA; lateral pixel size 150 nm; total imaging *z*-range 52.3 µm; axial step size 1.09 µm; pixel dwell time 0.49 µs.

Figure 2A. Pre-expansion: objective lens 10×/0.45 NA; lateral scan step (pixel) size 397 nm; total imaging *z*-range 100 µm; axial step size 2 µm; pixel dwell time 0.48 µs.

PlantEx: objective lens 10×/0.45 NA; lateral pixel size 241 nm; total imaging *z*-range 200 µm; axial step size 20 µm; pixel dwell time 0.4 µs with 4 scans per line.

Figure 2B. Pre-expansion: objective lens 40×/1.2 NA; lateral scan step (pixel) size 114 nm; total imaging *z*-range 25 µm; axial step size 1 µm; pixel dwell time 0.43 µs.

PlantEx: objective lens 20×/0.8 NA; lateral pixel size 149 nm; single plane; pixel dwell time 1.18 µs.

Figure 2C. Pre-expansion: objective lens 40×/1.2 NA; lateral scan step (pixel) size 114 nm; total imaging *z*-range 38 µm; axial step size 1 µm; pixel dwell time 0.43 µs.

PlantEx: objective lens 20×/0.8 NA; lateral pixel size 149 nm; total imaging *z*-range 62 µm; axial step size 2 µm; pixel dwell time 0.51 µs.

Figure 2D. See information from Fig. 1, panels (D), (E).

Figure 3A and Supplementary Fig. S4. Pre-expansion: objective lens 40×/1.2 NA; lateral scan step (pixel) size 100 nm; total imaging *z*-range 18 µm; axial step size 1 µm; pixel dwell time 0.48 µs.

PlantEx: objective lens 40×/1.2 NA; lateral pixel size 156 nm; total imaging *z*-range 40 µm; axial step size 1 µm; pixel dwell time 0.38 µs.

Figure 3B. Pre-expansion: objective lens 60×/1.2 NA; lateral pixel size 80 nm; pixel dwell time 25 µs, using the Abberior STED microscope in confocal mode.

PlantEx: objective lens 40×/1.2 NA; lateral pixel size 81 nm; total imaging *z*-range 41 µm; axial step size 1 µm; pixel dwell time 0.4 µs with 4 scans per line.

Figure 4A and B. PlantEx: confocal image; objective lens 60×/1.2 NA; pixel size 30 nm × 30 nm; pinhole size 0.83 Airy Unit; Excitation laser power: 7.2 µW; pixel dwell time 20 µs. PlantEx-STED: objective lens 60×/1.2 NA; pixel size 30 nm × 30 nm; pinhole size 0.83 AU; Excitation laser power: 7.2 µW; STED laser power: 32.5 mW; lateral/axial STED power ratio 100/0; pixel dwell time 63 µs; RESCue STED lower thresholds 1, 2, 5, and 10 counts after 21%, 28%, 52%, and 85% of total pixel dwell time, respectively; RESCue STED upper threshold 28 counts.

Figure 4E. PlantEx: confocal image; objective lens 60×/1.2 NA; pixel size 100 nm × 100 nm; pinhole size 1 AU; Excitation laser power: 5.1 µW; pixel dwell time 25 µs. PlantEx-STED: objective lens 60×/1.2 NA; pixel size 100 nm × 100 nm; pinhole size 1 AU; Excitation laser power: 5.8 µW; STED laser power: 4 mW; lateral/axial STED power ratio 0/100; pixel dwell time 25 µs; 2 line accumulations; RESCue STED lower thresholds 1, 2, 5, and 10 counts after 21%, 28%, 52%, and 85% of total pixel dwell time, respectively; RESCue STED upper threshold 28 counts.

Figure 4G–I. PlantEx-STED: objective lens 60×/1.2 NA; voxel size 100 nm × 100 nm × 100 nm; pinhole size 0.83 AU; Excitation laser power: 7.2 µW; STED laser power: 21.7 mW; lateral/axial STED power ratio 0/100; pixel dwell time 63 µs; RESCue STED lower thresholds 1, 2, 5, and 10 counts after 21%, 28%, 52%, and 85% of total pixel dwell time, respectively; RESCue STED upper threshold 28 counts.

Figure 4J. PlantEx-STED: objective lens 60×/1.2 NA; voxel size 100 nm × 100 nm × 100 nm; pinhole size 1 AU; Excitation laser power: 14.2 µW; STED laser power: 30 mW; lateral/axial STED power ratio 0/100; pixel dwell time 60 µs; volume size 10 × 10 × 80 µm^3^; *xyz* scan mode; pinhole 1 airy unit; RESCue STED lower thresholds 1, 2, 5, and 10 counts after 21%, 28%, 52%, and 85% of total pixel dwell time, respectively; RESCue STED upper threshold 28 counts.

Figure 5A. Spinning disc confocal image (Andor Dragonfly), objective lens 20×/0.75 NA; pinhole size 25 µm; lateral pixel size 300 nm; total imaging *z*-range 73 µm; axial step size 3 µm; 488 nm laser power: 14%; exposure time 100 ms, Andor Zyla sCMOS camera. Software: Andor Fusion version 2.2.0.49.

Figure 5B. PlantEx-STED: objective lens 60×/1.2 NA; pixel size 30 nm × 30 nm; pinhole size 1 AU; Excitation laser power 488 nm: 1.9 µW; 560nm: 3.3 µW; 640 nm: 14.2 µW; STED laser power: 10 mW; lateral/axial STED power ratio 100/0; pixel dwell time 60 µs.

Figure 5C. PlantEx-STED: objective lens 60×/1.2 NA; pixel size 30 nm × 30 nm; pinhole size 1 AU; Excitation laser power 488 nm: 5.6 µW; 560 nm: 6.6 µW; 640 nm: 14.2 µW; STED laser power: 10 mW; lateral/axial STED power ratio 100/0; pixel dwell time 40 µs.

Figure 5D. PlantEx-STED: objective lens 60×/1.2 NA; pixel size 30 nm × 30 nm; pinhole size 1 AU; Excitation laser power 560 nm: 5.3 µW; 640 nm: 1.4 µW; STED laser power: 5 mW; lateral/axial STED power ratio 100/0; pixel dwell time 50 µs.

Figure 5E. PlantEx-STED: objective lens 60×/1.2 NA; voxel size 100 nm × 100 nm × 100 nm; pinhole size 1 AU; Excitation laser power: 14.2 µW; STED laser power: 30 mW; lateral/axial STED power ratio 0/100; pixel dwell time 60 µs; Scan mode *xzy*; RESCue STED lower thresholds 1, 2, 5, and 10 counts after 21%, 28%, 52%, and 85% of total pixel dwell time, respectively; RESCue STED upper threshold 28 counts.

Supplementary Figure S3(A), (B). Pre-expansion: objective lens 40×/1.2 NA; lateral pixel size 100 nm, total imaging *z*-range 8.4 µm, axial step size 0.3 µm; pixel dwell time 0.59 µs. PlantEx: dataset 1: objective lens 40×/1.2 NA; lateral pixel size 100 nm; total imaging *z*-range 23 µm; axial step size 1 µm; pixel dwell time 1.31 µs. PlantEx: dataset 2: objective lens 40×/1.2 NA; lateral pixel size 100 nm; total imaging *z*-range 34 µm; axial step size 1 µm; pixel dwell time 1.31 µs.

Supplementary Figure S5. Live confocal imaging; lateral pixel size 104 nm, total imaging *z*-range 13 µm, axial step size 0.2 µm, pixel dwell time 0.34 µs.

Supplementary Figure S6. PlantEx: confocal image; lateral pixel size 100 nm × 100 nm; pinhole size 0.6 AU; Excitation laser power: 21.6 µW; pixel dwell time 20 µs. PlantEx-STED: pixel size 25 nm × 25 nm; pinhole size 0.6 AU; Excitation laser power: 21.6 µW; STED laser power: 21.7 mW; lateral/axial STED power ratio 100/0; pixel dwell time 20 µs.

### Image analysis

#### Processing of images

Images represent raw confocal or STED imaging data. No deconvolution or image restoration was applied. Intensity lookup tables, maximum intensity projections and color-coded projections were applied with FIJI/ImageJ, version 1.51p, 1.52p, 1.54f, or 1.53t. For further image processing and for clustering of vesicles, we used Python 3.6-3.10 together with the scikit-image (Van Der Walt et al. 2014) (ver. 0.20.0) library.

#### Image registration, determination of expansion factor, and distortion analysis

Distortion analysis was adapted from previously published methods (Sun et al. 2021) (https://github.com/Yujie-S/Click-ExM_data_process_and_example/tree/master). Pre- and post-expansion volumes were imaged with a confocal microscope, allowing quantification of distortions above the diffraction limit (∼200 nm). The BigWarp (Bogovic et al. 2016) Fiji-plugin (v. 7.0.7) was used for registration of corresponding pre- and post-expansion imaging volumes. First, we placed ∼20 landmarks at corresponding plant features in pre- and post-expansion imaging volumes. We then applied a similarity transformation (isotropic scaling, translation, and rotation) to the pre-expansion volume to overlap landmarks and align to the fixed post-expansion volume and crop to the same axial extent of the volumes. The expansion factor was extracted as the linear scaling factor of the similarity transformation minimizing squared landmark residuals using the script https://github.com/danzllab/CATS/tree/master/rcats_image-analysis/bigwarp. For Fig. 2B, we placed landmarks in 2D, as the post-expansion image was recorded as a single optical slice. For scaling, we either used the expansion factor determined for the specific experiment or the average expansion factor determined from *n* = 12 specimens (Fig. 1C). We then created maximum intensity projections from the aligned pre-/post-expanded volumes, selecting axial ranges for intensity projections to cover similar ranges in the sample, as judged by visual inspection. To make resolution and appearance of pre- and post-expansion images similar, we smoothed them with 2D Gaussian filters of different sizes (*σ* = 1 and *σ* = 6 pixels, respectively).

For the distortion analysis in Fig. 2A, we separated the foreground signal from the background: First, the pre-processed pre- and post-expansion projections were background-subtracted using a strong 2D Gaussian blur (*σ* = 80). Resulting negative values were clipped to zero. Then, we created signal-containing foreground masks by thresholding. A mask from pre- or post-expansion images was either applied directly or they were combined into a single binary mask by a logical OR operation. Finally, we applied a morphological closing operation to remove small, spurious holes in the mask. Thresholds and sizes of the closing operation were chosen by visual inspection.

To quantify nonlinearities in the expansion procedure, we computed distortion vector fields from the pre- and post-expansion images using the *imregdemons.m* function in MATLAB (version R2022b, MathWorks). We calculated the measurement error across different measurement lengths, as previously described (Chen et al. 2015). For this, we randomly sampled 200,000 pairs of feature points from the segmented foreground. The distortion field is applied to each pair to retrieve its corresponding transformed vector. We then calculated the measurement errors for different measurement lengths by subtracting corresponding feature vectors and taking the length of the difference vectors. For Fig. 2E, the RMS of measurement errors was calculated across the *n* = 4 specimens in Fig. 2A–D. For all figures, the RMS errors, measurement errors, and measurement lengths are shown in the pre-expansion scale.

#### Line profiles

Line profiles were created with FIJI/ImageJ, version 1.51n and 1.54f. Line profiles were created from single confocal or STED image planes. For noise reduction, averaging of 3-pixel width perpendicular to the direction of the line profile was applied.

#### Vesicle FWHM quantification in 2D

In the raw PlantEx-STED images, we first normalized the raw photon counts from 16-bit unsigned integer values to floating point values by dividing by the 99.9 percentile of the raw brightness values. We then applied an isotropic 2D Gaussian filter with a sigma of size 3.3 pixel (100 nm in the expanded sample) followed by the detection of local maxima in 2D. To suppress noisy background detections, we filtered for maxima having a brightness value greater than 25% of the maximum value in the image. We then selected isolated vesicles that were separated by at least 30 pixels (900 nm in the expanded sample) from other detections. We fitted an isotropic Gaussian function to these isolated vesicles. For the fitting, we extracted a square subimage of 41-pixel width (1.23 µm after expansion) centered at each isolated vesicle from the raw data. In each subimage, we fitted a Gaussian with 4 degrees of freedom (amplitude, isotropic standard deviation, center subpixel position in 2 spatial dimensions) or 5 degrees of freedom (amplitude, standard deviation in 2 directions, center subpixel position in 2 spatial dimensions) by minimizing squared residuals using the Levenberg–Marquardt algorithm. Upon convergence, we calculated the FWHM by multiplying the standard deviation with $2\sqrt{2\;\ln\;2\;\;}∼\;2.355$ and used the measured expansion factor to convert to the native tissue scale. We manually filtered the vesicles where the 2D Gaussian fitting failed due to background noise, overlap of neighboring peaks or signal from out-of-focus planes. In total, *n* = 74 vesicles across *n* = 3 samples were analyzed. Gaussian fitting was done using the Scipy library (ver. 1.12.0). We implemented the described routine as a Python command line script *gaussian_fit_fwhm* for 2D and 3D image data. For the vesicle FWHM estimate, we calculated a median value with its 25th and 75th percentile.

#### Vesicle FWHM quantification in 3D

In the raw volumetric PlantEx-STED images, we first normalized the raw photon counts from 16-bit unsigned integer values to floating point values by dividing by the 99.9 percentile of the raw brightness values. We then applied an isotropic 3D-Gaussian filter with a sigma of 1 voxel (100 nm in the expanded sample) followed by the detection of local maxima in 3D. To suppress noisy background detections, we filtered for local maxima having a brightness value greater than 25% of the maximum value in the image. We then selected isolated vesicles that were separated by at least 18 voxels (1.8 µm after expansion) from other detections and fitted an isotropic 3D-Gaussian function to these. For the fitting, we extracted cubes of 21 voxel edge length (2.1 µm after expansion) centered at each isolated vesicle from the raw data. In each subvolume, we fitted a 3D-Gaussian with 5 degrees of freedom (amplitude, isotropic standard deviation, and center subpixel positions in 3 spatial dimensions) or 7 degrees of freedom (amplitude, standard deviation in 3 directions, center subpixel position in 3 spatial dimensions) by minimizing squared residuals using the Levenberg–Marquardt algorithm. Upon convergence, we calculated the FWHM and used the measured expansion factor to convert to the native tissue scale. In total, *n* = 112 vesicles were analyzed across *n* = 3 specimens.

#### Identification and visualization of 3D clusters

In the raw PlantEx-STED images, we first normalized the raw photon counts from 16-bit unsigned integer values to floating point values by dividing by the 99.9 percentile of the raw brightness values. We then applied an isotropic Gaussian filter with a sigma of 0.7 pixel (70 nm after expansion), followed by the detection of local maxima in 3D. The minimum allowed separation between any 2 local maxima was set to 2 voxel edge lengths (200 nm). If 2 local maxima were closer, we chose the maximum with the higher brightness value. To suppress noisy background detections, we filtered for maxima with a brightness of at least 10% of the maximum value in the image. In order to identify clusters of sec21-positive puncta, we applied the ordering points to identify the clustering structure (OPTICS) algorithm (Ankerst et al. 1999). We set the input parameter for the minimum cluster size to 10 (minimum number of vesicles to form a cluster). All other parameters were left to their default values (scikit-learn ver. 0.21.3). 3D rendering of vesicles was done using the SciView library (Günther et al. 2019) in FIJI/ImageJ, version 1.51p.

### Replication

In all images, representative data from individual experiments are shown. As this manuscript reports on a technological development, a number of experiments with some variation of parameters have been performed, including during the development phase. Stated replicates give a lower bound on how many times individual experiments were performed with similar results. For high-quality representation of tissue structure, optimum fixation, labeling, tissue preservation, and expansion are required (see the “Discussion” section). For analysis, only datasets of high labeling, expansion, and imaging quality were pursued and datasets of lower quality were discarded. The sample size and number of biological replicates for quantifications are given in the respective figure captions.

### Accession numbers

Sequence data from this article can be found in the GenBank/EMBL data libraries under accession numbers: *VHA-*a1: AT2G28520; MAP4: M72414 (Marc et al. 1998), tubulin, alpha 1: AT1G64740, alpha 2: AT1G50010, alpha 3: AT5G19770, alpha 4: AT1G04820, alpha 5: AT5G19780, alpha 6: AT4G14960, beta 1: AT1G75780, beta 2: AT5G62690, beta 3: AT5G62700, beta 4: AT5G44340, beta 5: AT1G20010, beta 6: AT5G12250, beta 7: AT5G12250, beta 8: AT5G23860, beta 9: AT4G20890; *sec21* coatomer subunit gamma: AT4G34450; PIN1: AT1G73590; PIN2: AT5G57090.

## Supplementary Material

koaf006_Supplementary_Data

## Data Availability

The data that support the findings of this study are available via ISTA's data repository at https://doi.org/10.15479/AT:ISTA:18837.

## Supplementary data

The following materials are available in the online version of this article.

**Supplementary Figure S1.** Nonspecific proteolytic activity of cell-wall digestion cocktail.

**Supplementary Figure S2.** Alignment of samples for determination of expansion factor and distortions.

**Supplementary Figure S3.** Mitotic figures in tubulin-labeled PlantEx samples.

**Supplementary Figure S4.** Example of immunolabeling with cell-to-cell variability.

**Supplementary Figure S5.** Live confocal imaging in MAP4-GFP-expressing *A. thaliana* root.

**Supplementary Figure S6.** STED imaging in *A. thaliana* root without PlantEx.

**Supplementary Figure S7.** FWHM of COPI-coated vesicles in PlantEx-STED microscopy.

**Supplementary Figure S8.** 3D-rendering of COPI-coated vesicle distribution imaged with PlantEx-STED microscopy.

**Supplementary Figure S9.** PlantEx pan-protein labeling with noninverted intensity lookup table.

**Supplementary Video S1.** COPI-coated vesicles (green) and TGN (magenta) in conventional confocal microscopy before expansion.

**Supplementary Video S2.** COPI-coated vesicles (green) and trans-Golgi network (magenta) with PlantEx, imaged with confocal microscopy.

**Supplementary Video S3–S6.** COPI-coated vesicles in tissue volumes imaged with PlantEx-STED.

**Supplementary Video S7–S10.** Movies of the 3D renderings of COPI-coated vesicles imaged with PlantEx-STED at near-isotropic resolution.

## Code availability

The custom-written code used and described in this manuscript is available via Github (https://github.com/danzllab/PlantEx).

## Dive Curated Terms

The following phenotypic, genotypic, and functional terms are of significance to the work described in this paper:

VHA-a1 Gramene: AT2G28520
VHA-a1 Araport: AT2G28520

SEC21 Gramene: AT4G34450
SEC21 Araport: AT4G34450

TGN Gramene: trans-Golgi network
TGN Araport: trans-Golgi network

PIN2 Gramene: AT5G57090
PIN2 Araport: AT5G57090
