# Supplementary material for: Super-resolution expansion microscopy in plant roots
Source: Plant Cell. 2025 Jan 10;37(4):koaf006. doi: 10.1093/plcell/koaf006 (PMC11983393; doi:10.1093/plcell/koaf006)
Supplement: koaf006_Supplementary_Data [file koaf006_supplementary_data.zip › TPC2024BR00212R1_Supplementary Video Legends_20241223.docx]

## Supplementary Video Legends

*Supplementary Video 1.* COPI-coated vesicles (green) and trans-Golgi network (magenta) in conventional confocal microscopy before expansion. Pre-expansion image stack from the expanded sample in Fig. 3b.

*Supplementary Video 2.* COPI-coated vesicles (green) and trans-Golgi network (magenta) with PlantEx, imaged with confocal microscopy. Image stack of a larger region from the same expanded sample as in Fig. 3b.

*Supplementary Videos 3-6*. COPI-coated vesicles in tissue volumes imaged with PlantEx-stimulated emission depletion (STED). Fly-through of the raw imaging data along the optical axis for individual tissue volumes displayed in Fig. 4g and Suppl. Fig. S8. Scale bars refer to original tissue scale.

*Supplementary Videos 7-10*. Movies of the 3D renderings of COPI-coated vesicles imaged with PlantEx-stimulated emission depletion (STED) at near-isotropic resolution. Same tissue volumes as in Fig. 4g and Suppl. Fig. S8. First the imaging data is shown, then in addition vesicle detections. Next, they are classified into vesicles assigned to clusters (green) and those outside clusters (gray) and subsequently only the clustered vesicles are shown. Clusters are then color coded.
