# Supplementary material for: Super-resolution expansion microscopy in plant roots
Source: Plant Cell. 2025 Jan 10;37(4):koaf006. doi: 10.1093/plcell/koaf006 (PMC11983393; doi:10.1093/plcell/koaf006)
Supplement: koaf006_Supplementary_Data [file koaf006_supplementary_data.zip › TPC2024-BR-00212R1_Supplementary_Information.pdf]

Supplementary Information

Super-resolution expansion microscopy in plant roots

Short title: Expansion microscopy in plants

Michelle Gallei[1]†, Sven Truckenbrodt[1,2]†, Caroline Kreuzinger[1], Syamala Inumella[1], Vitali Vistunou[1], Christoph Sommer[1], Mojtaba R. Tavakoli[1], Nathalie Agudelo Dueñas[1], Jakob Vorlaufer[1], Wiebke Jahr[1], Marek Randuch[1], Alexander Johnson[1,3], Eva Benková[1], Jiří Friml[1], Johann G. Danzl[1]

[1]Institute of Science and Technology Austria, Am Campus 1, 3400 Klosterneuburg, Austria
[2]present address: E11 Bio, Alameda, CA, USA
[4]present address: Biosciences, University of Exeter, Exeter EX4 4QD, UK

†authors contributed equally
Correspondence to: Johann G. Danzl (johann.danzl@ista.ac.at)

Contents

Supplementary Figure S1: Non-specific proteolytic activity of cell wall digestion cocktail. 2

Supplementary Figure S2: Alignment of samples for determination of expansion factor and distortions. 3

Supplementary Figure S3: Mitotic figures in tubulin-labeled PlantEx samples. 4

Supplementary Figure S4: Example of immunolabeling with cell-to-cell variability. 5

Supplementary Figure S5: Live confocal imaging in MAP4-GFP expressing *A. thaliana* root. 6

Supplementary Figure S6: STED imaging in *A. thaliana* root without PlantEx. 7

Supplementary Figure S7: FWHM of COPI-coated vesicles in PlantEx-STED microscopy. 8

Supplementary Figure S8: 3D-rendering of COPI-coated vesicle distribution imaged with PlantEx-STED microscopy. 9

Supplementary Figure S9: PlantEx pan-protein labeling with non-inverted intensity lookup table. 10

Supplementary Figure S1: Non-specific proteolytic activity of cell wall digestion cocktail.

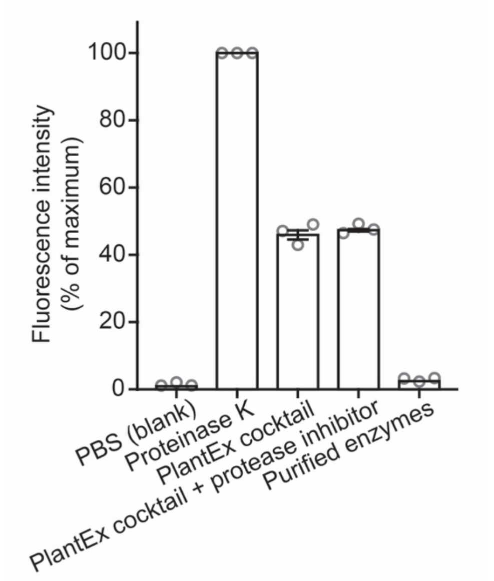

Non-specific proteolytic activity of cell wall digestion cocktail. Fluorescence assay for non-specific proteolytic activity (EnzChek, Thermo Fisher), with fluorescence readout intensity normalized to proteinase K activity. Concentrations, incubation times, and temperatures for proteinase K and PlantEx cell wall digestion cocktail were chosen identical to the respective digestion steps in PlantEx experiments. The PlantEx cell wall digestion cocktail contains natural products that are not chemically defined (fungal extracts) and displayed 46 ± 3 % (mean ± s.d.) of non-specific proteolytic activity relative to proteinase K. Proteolytic activity was not suppressed by a proprietary protease inhibitor mix (PlantEx cocktail + protease inhibitor, cOmplete, Sigma Aldrich). A mixture of purified enzymes targeting specific cell wall components (α-amylase, α-L-arabinofuranosidase, β-mannanase, cellulase, pectate lyase, xyloglucanase) we tested for PlantEx did not display substantial proteolytic activity but was not effective at achieving mechanical homogenization for expansion. PBS: phosphate-buffered saline. Mean ± s.d., datapoints represent 3 experimental repetitions. Supports Figure 1.

Supplementary Figure S2: Alignment of samples for determination of expansion factor and distortions.

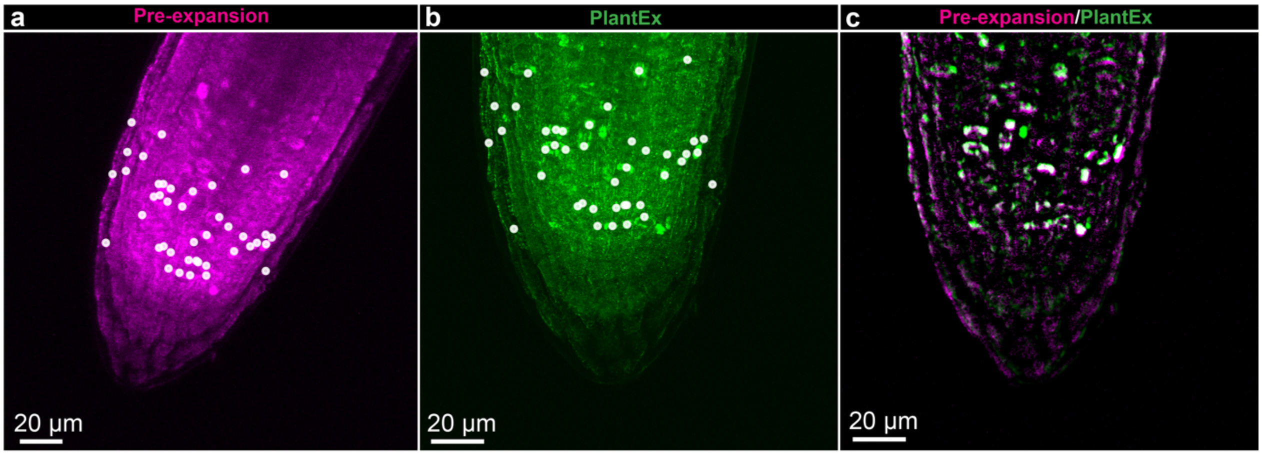

Alignment of samples for determination of expansion factor and distortions. **a,b,** Manually placed landmarks (white) overlaid with a maximum intensity projection of the pre- and post-expansion confocal imaging stacks in Fig. 2a featuring labeling for tubulin. For alignment, landmark features that were readily identified in both pre-expansion and PlantEx image stacks were marked using the BigWarp plugin for ImageJ. Note that the landmarks were not actually set on the shown maximum intensity projections, but on individual slices of the image stack, to maximize alignment fidelity. For alignment of pre-expansion and PlantEx images, a (linear) similarity transformation with the following 7 degrees of freedom was performed: isotropic scaling (equivalent to expansion factor), rotation (3 angles), and 3D-translation (3 axes). Scale bars: 20 µm (corresponding to 82 µm in expanded sample, expansion factor exF=4.1). **c**, Overlay of pre- and post-expansion images. The homogeneous signal component was removed by Gaussian background subtraction to focus distortion analysis on distinct image features. Supports Figures 1 and 2.

Supplementary Figure S3: Mitotic figures in tubulin-labeled PlantEx samples.

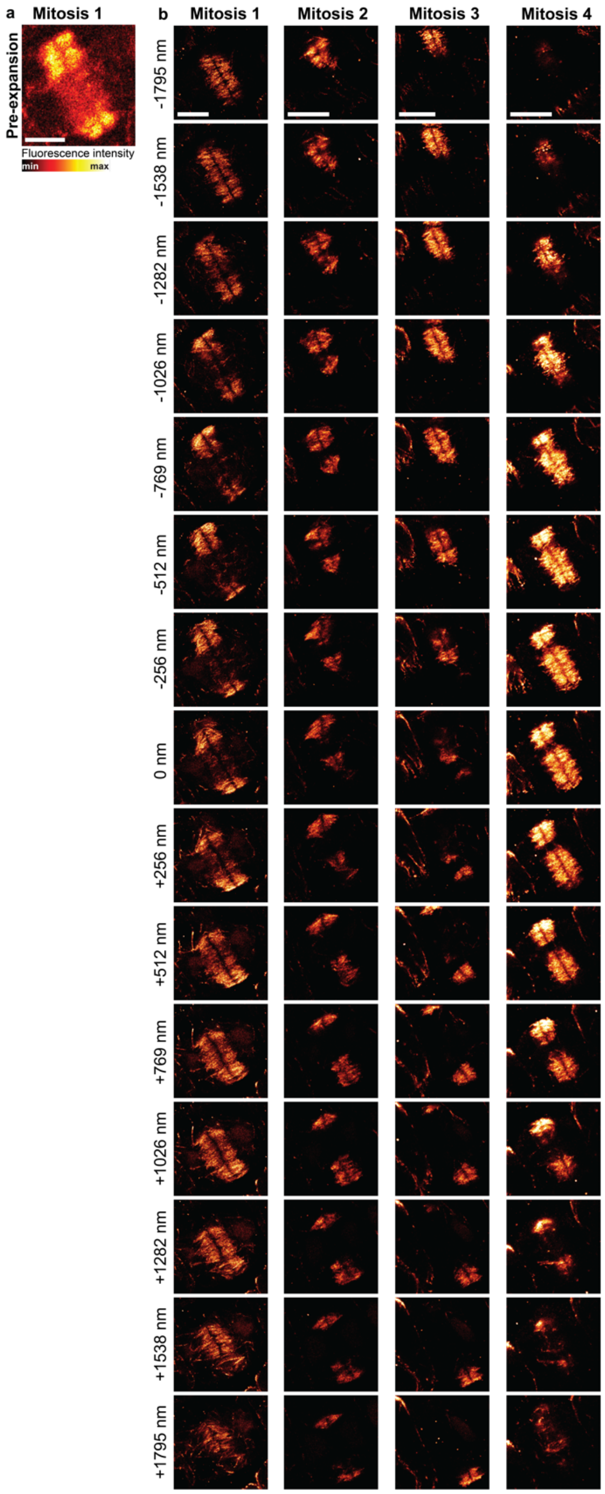

Mitotic figures in tubulin-labeled PlantEx samples. **a,** Confocal image from a non-expanded *A. thaliana* root sample immuno-labeled for tubulin. Sample was treated with taxol prior to fixation to increase number of mitotic arrests. **b,** Confocal image stacks in the same root after application of PlantEx, showing the same mitotic figure at 4-fold increased resolution (mitosis 1) and 3 further examples from the same specimen. Scale bars: 5 µm. Supports Figure 1.

Supplementary Figure S4: Example of immunolabeling with cell-to-cell variability.

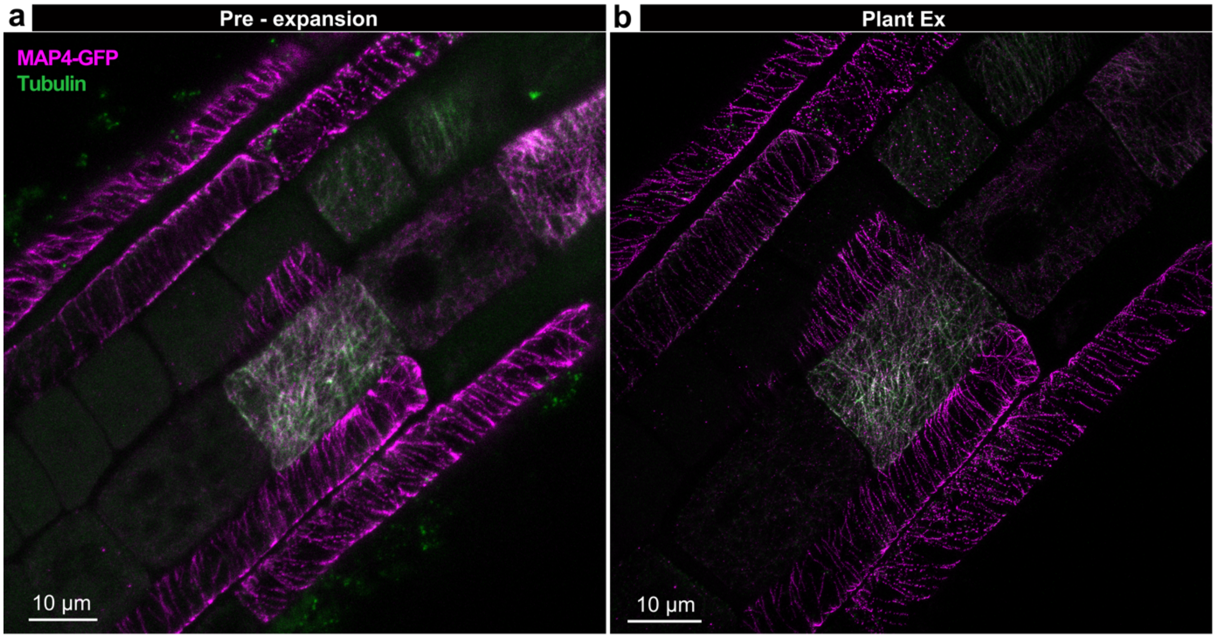

Example of immunolabeling with cell-to-cell variability. a, Pre-expansion confocal overview image in the same specimen as in Fig. 3a, in an Arabidoposis line expressing a microtubule associated protein 4 - green fluorescent protein (MAP4-GFP) fusion protein. GFP and tubulin were detected via immunolabeling. Here, antibody labeling was variable between cells, which may occur and likely reflects variability between cells in antibody access in the pre-expansion immunolabeling. **b,** Confocal image of the same region acquired after expansion. Maximum intensity projections covering approximately equal axial ranges as in Fig. 3a. Supports Figure 3.

Supplementary Figure S5: Live confocal imaging in MAP4-GFP expressing *A. thaliana* root.

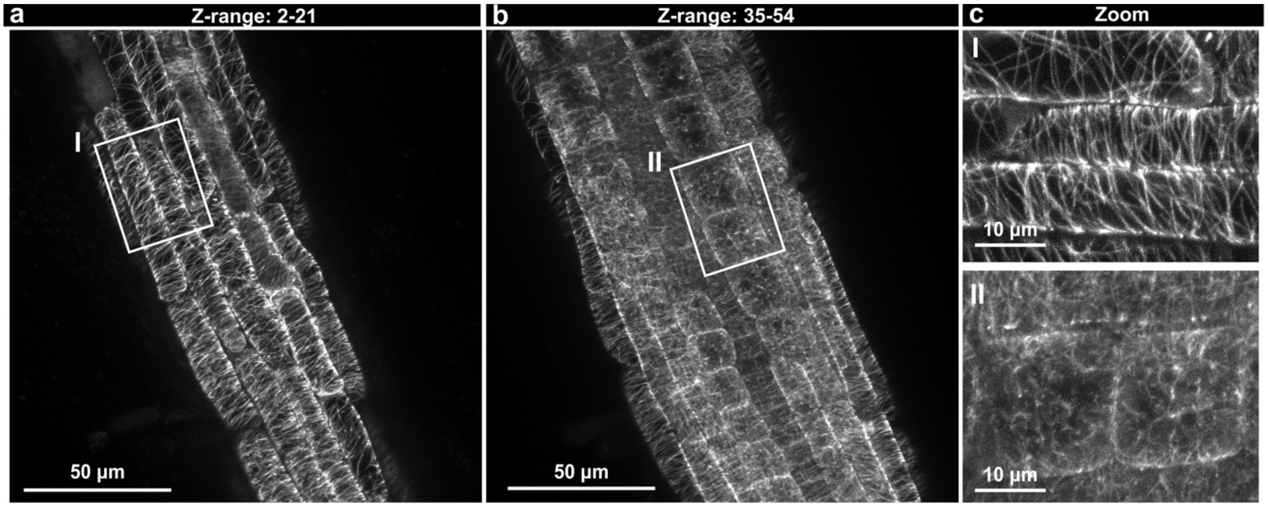

Live confocal imaging in MAP4-GFP expressing *A. thaliana* root. a, Maximum intensity projection of a confocal imaging stack acquired with a high-NA water immersion objective lens in the most superficial layer of the root of a 5-day old transgenic seedling expressing a microtubule associated protein 4 - green fluorescent protein (MAP4-GFP) fusion protein. The projection covers ~4 µm axial imaging extent. Numbers for the *z*-range refer to confocal imaging planes, spaced 200 nm. **b,** Similar maximum intensity projection starting ~7 µm deep in the specimen. **c,** Enlarged view of the boxed regions in panels a and b. Axial extent of the maximum intensity projection is similar as in Fig. 3a. Supports Figure 3.

Supplementary Figure S6: STED imaging in *A. thaliana* root without PlantEx.

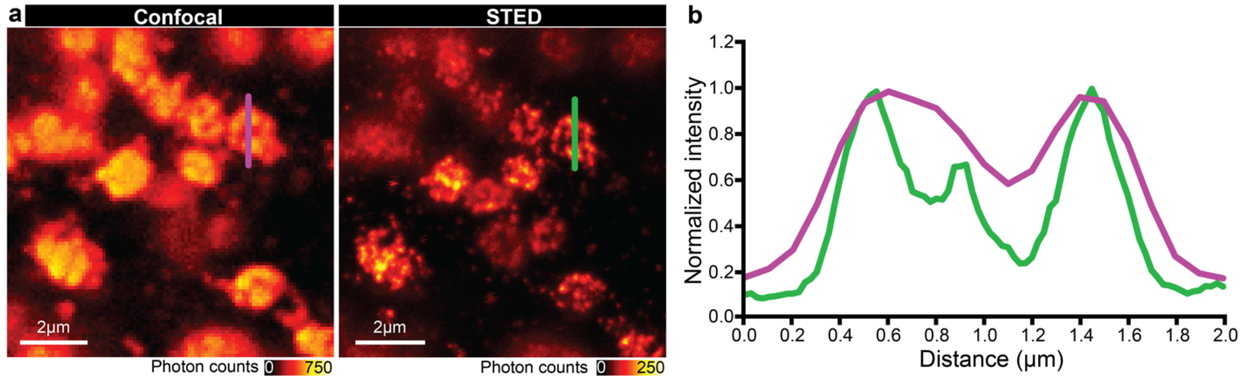

Stimulated emission depletion (STED) imaging in *A. thaliana* root without PlantEx. a, *A. thaliana* root tip immuno-labeled for Sec21 (γ-subunit of the coatomer in COPI-coated vesicles (γ-COP)) and imaged with confocal and STED microscopy (with lateral resolution increase, *xy*-STED pattern), respectively. No hydrogel expansion was applied. Scale bar: 2 µm. **b,** Line profiles as indicated in panel a. *A. thaliana* roots are challenging samples for STED imaging, due to factors including scattering and refractive index mismatch and variation. Supports Figure 4.

Supplementary Figure S7: FWHM of COPI-coated vesicles in PlantEx-STED microscopy.

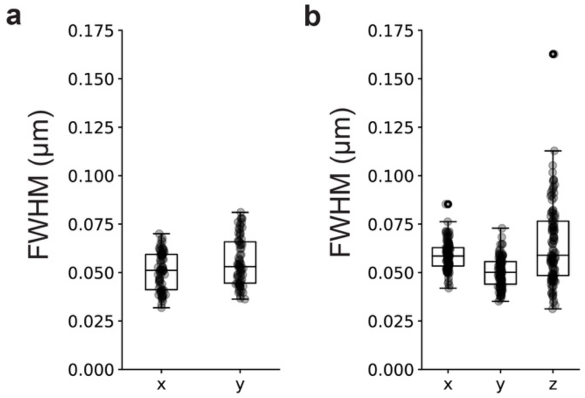

Full-width-at-half-maximum (FWHM) of COPI-coated vesicles in PlantEx-stimulated emission depletion (STED) microscopy. **a,** Anisotropic 2D Gaussian fits (5 degrees of freedom: amplitude, width in 2 directions, position in 2D) of the same data as in Fig. 4d. FWHM median (lower, upper quartiles) of the 74 vesicles analyzed across *n*=3 specimens: *x*: 51 nm (41 nm, 59 nm); *y*: 53 nm (45 nm, 66 nm). **b,** Anisotropic 3D Gaussian fits (7 degrees of freedom: amplitude, width in 3 directions, position in 3D) to the same data as in Fig. 4f. FWHM median (lower, upper quartile) of the 112 vesicles analyzed across *n*=3 specimens: *x*: 59 nm (53 nm, 63 nm); *y*: 50 nm (44 nm, 56 nm); *z*: 59 nm (48 nm, 77 nm). Here, application of a *z*-STED pattern with resolution increase predominantly in the axial direction leads to near-isotropic overall resolution. Box plot centre lines, median; box limits, upper and lower quartiles; whiskers, 1.5x interquartile range; points, individual data points. Supports Figure 4.

Supplementary Figure S8: 3D-rendering of COPI-coated vesicle distribution imaged with PlantEx-STED microscopy.

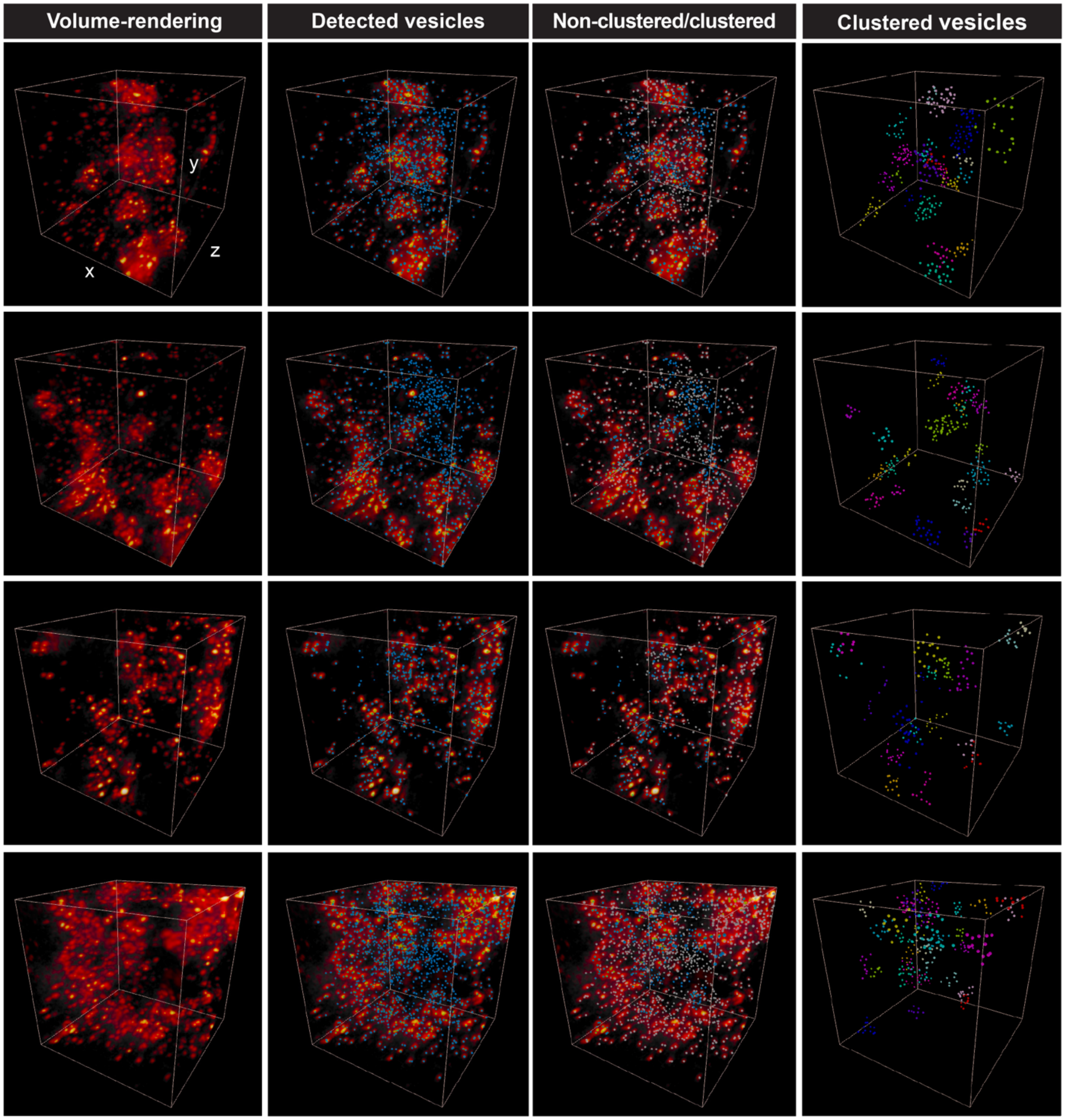

3D-rendering of COPI-coated vesicle distribution imaged with PlantEx-stimulated emission depletion (STED) microscopy. Volume renderings from a PlantEx *Arabidopsis thaliana* whole-mount root sample immunostained for COPI-coated vesicles (γ-COP, sec21) and imaged with near-isotropic STED microscopy (*z*-STED pattern to increase resolution predominantly in the axial direction). Imaging volumes were 15 x 15 x 15 μm^3 after expansion, corresponding to 3.8 x 3.8 x 3.8 μm^3 in the native tissue (column 1). For visualization, γ-COP positive puncta were detected as local intensity maxima (column 2) and classified as clustered (green) or non-clustered (grey) by the ordering points to identify the clustering structure (OPTICS) algorithm (Ankerst et al., 1999) (column 3), with a minimum cluster size of 10. The last column shows only detections assigned to clusters, with individual clusters color coded. Imaging volumes contained 925, 1066, 548, and 1497 detections in the 4 imaging volumes from top to bottom. Supports Figure 4.

Supplementary Figure S9: PlantEx pan-protein labeling with non-inverted intensity lookup table.

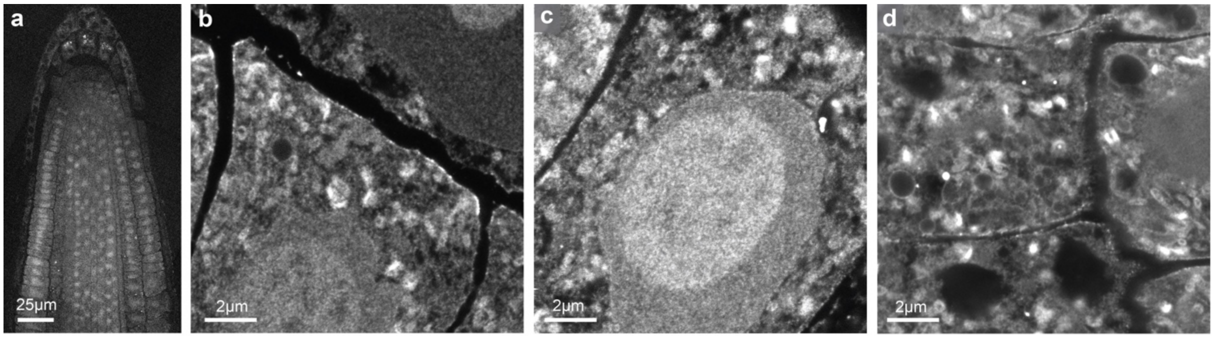

PlantEx pan-protein labeling with non-inverted intensity lookup table. Same data as in Fig. 5a-d, without inverting the intensity lookup table. Black: low protein density. White: high protein density. Supports Figure 5.

References

Ankerst, M., Breunig, M.M., Kriegel, H.P., Sander, J., 1999. OPTICS: Ordering Points to Identify the Clustering Structure. SIGMOD Record (ACM Special Interest Group on Management of Data) 28, 49–60. https://doi.org/10.1145/304181.304187
